# Therapeutic efficacy of AAV-mediated restoration of PKP2 in arrhythmogenic cardiomyopathy

**DOI:** 10.1038/s44161-023-00378-9

**Published:** 2023-12-07

**Authors:** Eirini Kyriakopoulou, Danielle Versteeg, Hesther de Ruiter, Ilaria Perini, Fitzwilliam Seibertz, Yannic Döring, Lorena Zentilin, Hoyee Tsui, Sebastiaan J. van Kampen, Malte Tiburcy, Tim Meyer, Niels Voigt, van J. Peter Tintelen, Wolfram H. Zimmermann, Mauro Giacca, Eva van Rooij

**Affiliations:** 1 Hubrecht Institute-KNAW and Utrecht University Medical Center, Utrecht, the Netherlands; 2 Institute of Pharmacology and Toxicology, University Medical Center Gottingen (UMG), Göttingen, Germany; 3 German Center for Cardiovascular Research (DZHK), partner site Göttingen, Göttingen, Germany; 4 Cluster of Excellence ‘Multiscale Bioimaging: from Molecular Machines to Networks of Excitable Cells’ (MBExC), University of Göttingen, Göttingen, Germany; 5 Nanion Technologies GmbH, Munich, Germany; 6 International Centre for Genetic Engineering and Biotechnology (ICGEB), Trieste, Italy; 7 Department of Genetics, University Medical Centre Utrecht, Utrecht, the Netherlands; 8 German Center for Neurodegenerative Diseases (DZNE), Göttingen, Germany; 9 Fraunhofer Institute for Translational Medicine and Pharmacology (ITMP), Göttingen, Germany; 10 British Heart Foundation Centre of Research Excellence, School of Cardiovascular Medicine & Sciences, King’s College London, London, UK; 11 Department of Cardiology, University Medical Center Utrecht, Utrecht, the Netherlands

**Keywords:** Cardiomyopathies, Genetic vectors

## Abstract

Arrhythmogenic cardiomyopathy is a severe cardiac disorder characterized by lethal arrhythmias and sudden cardiac death, with currently no effective treatment. Plakophilin 2 (*PKP2*) is the most frequently affected gene. Here we show that adeno-associated virus (AAV)-mediated delivery of PKP2 in *PKP2*^c.2013delC/WT^ induced pluripotent stem cell-derived cardiomyocytes restored not only cardiac PKP2 levels but also the levels of other junctional proteins, found to be decreased in response to the mutation. PKP2 restoration improved sodium conduction, indicating rescue of the arrhythmic substrate in *PKP2* mutant induced pluripotent stem cell-derived cardiomyocytes. Additionally, it enhanced contractile function and normalized contraction kinetics in *PKP2* mutant engineered human myocardium. Recovery of desmosomal integrity and cardiac function was corroborated in vivo, by treating heterozygous *Pkp2*^c.1755delA^ knock-in mice. Long-term treatment with AAV9–PKP2 prevented cardiac dysfunction in 12-month-old *Pkp2*^c.1755delA/WT^ mice, without affecting wild-type mice. These findings encourage clinical exploration of PKP2 gene therapy for patients with PKP2 haploinsufficiency.

## Abstract (web summary)

Kyriakopoulou et al. report that adeno-associated virus-mediated delivery of PKP2 in *PKP2*^c.2013delC/WT^ induced pluripotent stem cell-derived cardiomyocytes and heterozygous *Pkp2*^c.1755delA^ knock-in mice restores cardiomyocyte junctions, enhances cardiac function and mitigates arrhythmogenic substrates leading to arrhythmogenic cardiomyopathy.

## Main

Arrhythmogenic cardiomyopathy (ACM) is a progressive genetic cardiac disorder with a prevalence ranging from 1:2,000 to 1:5,000 (ref. ^1^). Early diagnosis is often hindered by phenotypic complexity and variable disease penetrance^2^. At the clinically concealed phase of the disease, patients often present asymptomatic or with mild electrocardiogram abnormalities, while possessing high risk of sudden cardiac death^3^. As disease progresses, structural remodeling characterized by fibro-fatty tissue infiltration within the myocardium becomes evident, ultimately leading to life-threatening ventricular arrhythmias and heart failure, a condition that often requires heart transplantation^3,4^.

Approximately 50% of patients with ACM carry genetic mutations in the desmosomal genes: plakophilin 2 (*PKP2*), plakoglobin (*JUP*), desmoplakin (*DSP*), desmocollin (*DSC2*) and desmoglein (*DSG2*)^5^. Desmosomes are robust multiprotein structures localized within the intercalated discs (IDs), where they facilitate mechanical coupling of the adjacent cardiomyocytes (CMs)^6^. Despite the classical notion that desmosomes function individually, a close connection and interaction of desmosomes with more ID components such as ion channels, gap junctions and adherens junctions has been described, together forming the area composita or connexome^6–9^.

*PKP2* is the most commonly affected gene in patients with ACM^10^. Specifically, mutations in *PKP2* have been strongly associated with the onset and development of arrhythmogenic right ventricular cardiomyopathy, a distinctive subtype of ACM marked by its pronounced impact on the right ventricle. While there is growing evidence revealing contributions from both the biventricular and left ventricular regions to the ACM phenotype, it is worth noting that arrhythmogenic right ventricular cardiomyopathy continues to be the predominant subtype within the ACM spectrum, primarily inherited through an autosomal dominant pattern^11^. The vast majority of genetic alterations affecting *PKP2* are truncating variants^12^. These mutant transcripts are often degraded by nonsense-mediated messenger RNA decay, causing PKP2 haploinsufficiency, which is an important pathogenic ACM driver^12–14^. The fundamental role of PKP2 in ACM disease pathogenesis and progression has been highlighted by several studies^9,14–22^. A study on a cardiomyocyte-specific, inducible deletion of *Pkp2* in mice revealed PKP2 as a crucial regulator of calcium cycling and cardiac rhythm^15^. These findings were further corroborated on a heterozygous *Pkp2* knockout mouse model exposed to environmental stress stimuli^22^. Another study demonstrated endogenous correlation of *PKP2* transcript abundance with the abundance of transcripts encoding inflammatory/immune response factors, the presence of which is thought to mediate ACM progression^17^. Interestingly, a study aiming to explore the pathogenic mechanisms leading to ventricular dilation and decreased systolic function associated with ACM, revealed that truncating *PKP2* mutations impair CM contractility by disrupting sarcomere stability and localization^14^. Of relevance, it was recently shown that cardiac levels of PKP2 directly correlate to protein levels of other desmosomal and adherens junction proteins in patients^20^. These data suggest that PKP2 fulfills a key anchoring role for the stabilization and function of other desmosomal and ID proteins. PKP2 loss as a consequence of truncating variants induces desmosomal instability and the eventual degradation of the area composita-related proteins, whereby downstream disease processes become activated.

Despite an improvement in our knowledge about the molecular triggers underlying ACM, targeted and effective therapeutic interventions for this disease remain lacking. While current treatment options are more focused on treating disease symptoms^23^, targeting the primary cause of disease consequently leading to ACM would have curative potential.

Here we show that adeno-associated virus (AAV)-mediated PKP2 restoration results in the re-formation of the desmosomal complex and consequently an improvement in contractile function in *PKP2*^c.2013delC/WT^ induced pluripotent stem (iPS) cell-derived CMs, *PKP2*^c.2013delC/WT^ engineered human myocardium (EHM) and *Pkp2* mutant knock-in mice. Molecular restoration of desmosomal and non-desmosomal protein components within the ID was observed following exogenous administration of *Pkp2*, which successfully prevented the functional decline induced by PKP2 haploinsufficiency. Of particular importance, the overexpression of *PKP2* in healthy cells and mice did not elicit alterations in desmosomal protein levels, nor did it induce a decline in cardiac function. These findings suggest that restoration of PKP2 levels in patients with ACM harboring a pathogenic *PKP2* mutation could lead to a therapeutic benefit.

## Results

### PKP2 restoration enhances CM function

In an effort to assess the relevance of PKP2 haploinsufficiency for the ACM population, we conducted a comprehensive analysis of the Dutch ACM registry (https://www.acmregistry.nl/). Of over 228 index patients from the registry, 137 (60%) have a (likely) pathogenic variant underlying their ACM phenotype^24^. Ninety-seven patients carry (likely) pathogenic *PKP2* variants. Thirty-four of these have 8 different single nucleotide substitutions in *PKP2* introducing a stop codon, originally described as c.235C>T; p.(Arg79*), c.258T>G; p.(Tyr86*), c.397C>T; p.(Gln133*), c.1848C>A; p.(Tyr616*), c.1951C>T; p.(Arg651*), c.2028G>A; p.(Trp676*), c.2203C>T; p.(Arg735*), c.2421C>A p.(Tyr807*)^25^. These findings highlight the importance of *PKP2* mutations, particularly nonsense mutations, in the pathogenesis and advancement of the disease.

**Fig. 1:**
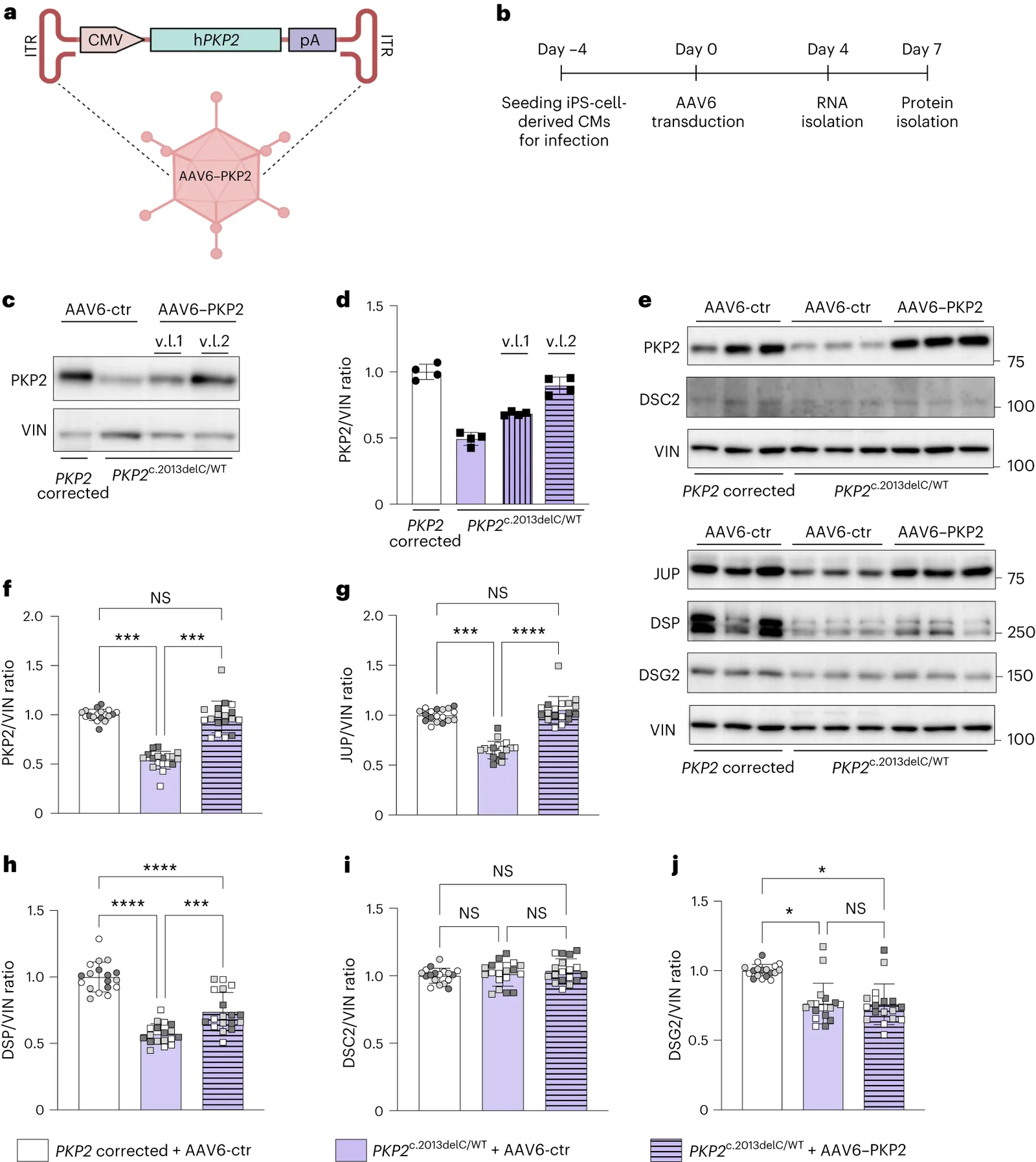
AAV-mediated restoration of PKP2 in *PKP2*^c.2013delC/WT^ mutant iPS-cell-derived CMs results in molecular rescue of desmosomal assembly. **a**, Graphical representation of the AAV expression cassette used in iPS-cell-derived CMs. ITR, Inverted Terminal Repeat Sequences. **b**, Timeline for AAV6–PKP2 transduction of iPS-cell-derived CMs. **c**, Representative immunoblots showing v.l.-dependent PKP2 protein levels in *PKP2*^c.2013delC/WT^ mutant cells in response to AAV6-PKP2. v.l.1 = 0.5 × 10^3^ v.g. per cell, v.l.2 = 5 × 10^3^ v.g. per cell. Vinculin (VIN) was used as a loading control. **d**, Quantification of **c**, *n* = 4 technical replicates per condition. **e**, Representative immunoblots for PKP2, JUP, DSP, DSG2 and DSC2 in corrected and mutant iPS-cell-derived CMs upon transduction with AAV6-ctr or AAV6–PKP2. VIN was used as a loading control. **f**, Quantification of PKP2 protein levels. **g**, Quantification of JUP protein levels. **h**, Quantification of DSP protein levels. **i**, Quantification of DSC2 protein levels. **j**, Quantification of DSG2 protein levels. Colored dots represent distinct CM differentiation, *n* = 6 technical replicates and 3 biological replicates per condition. Data is presented as mean values ± s.e.m. Statistical significance is derived from biological replicates and determined with one-way ANOVA (Tukey’s post-hoc test), *P* value at ****P < 0.0001, ***P < 0.001, **P < 0.01, *P < 0.05, and not significant (NS). *P* values (from left to right): 0.0003, 0.91, 0.0004 (**f**); 0.0002, 0.3778, <0.0001 (**g**); <0.0001, <0.0001, 0.0007 (**h**); 0.0138, 0.0125, 0.9955 (**j**). Source data

**Extended Data Fig. 1:**
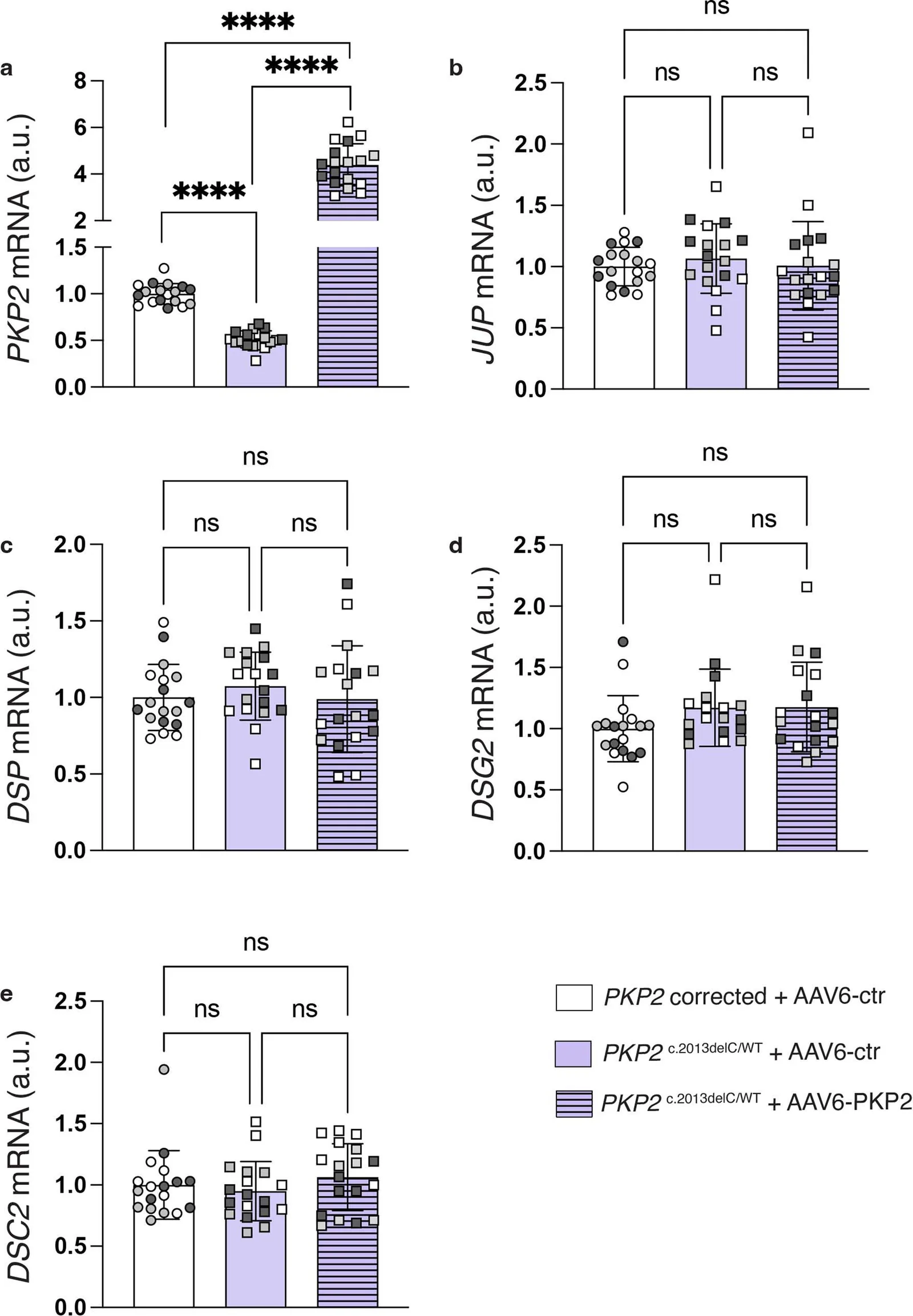
AAV-mediated restoration of PKP2 in *PKP2*^c.2013delC/WT^ mutant iPS-cell-derived CMs does not affect the mRNA levels of desmosomal proteins. **a-e**, Gene expression of *PKP2*, *JUP*, *DSP*, *DSG2* and *DSC2* in *Pkp2*^c.2013delC/WT^ iPS-cell-derived CMs upon transduction with either AAV6-ctr or AAV6-PKP2. As a reference, the *PKP2* corrected iPS-cell-derived CM line was transduced with AAV6-ctr. Values were normalized to *GUS*. n = 3 biological replicates (distinct CM differentiations) and 6 technical replicates per differentiation. Data is presented as mean +/− s.e.m. Statistical significance is derived from biological replicates and is determined with one-way ANOVA (Tukey’s post-hoc test), p-value at ****P < 0.0001, ***P < 0.001, **P < 0.01, *P < 0.05, and not significant (ns). Exact *P*-value for panel (a) is < 0.0001 for all comparisons. Source data

**Extended Data Fig. 2:**
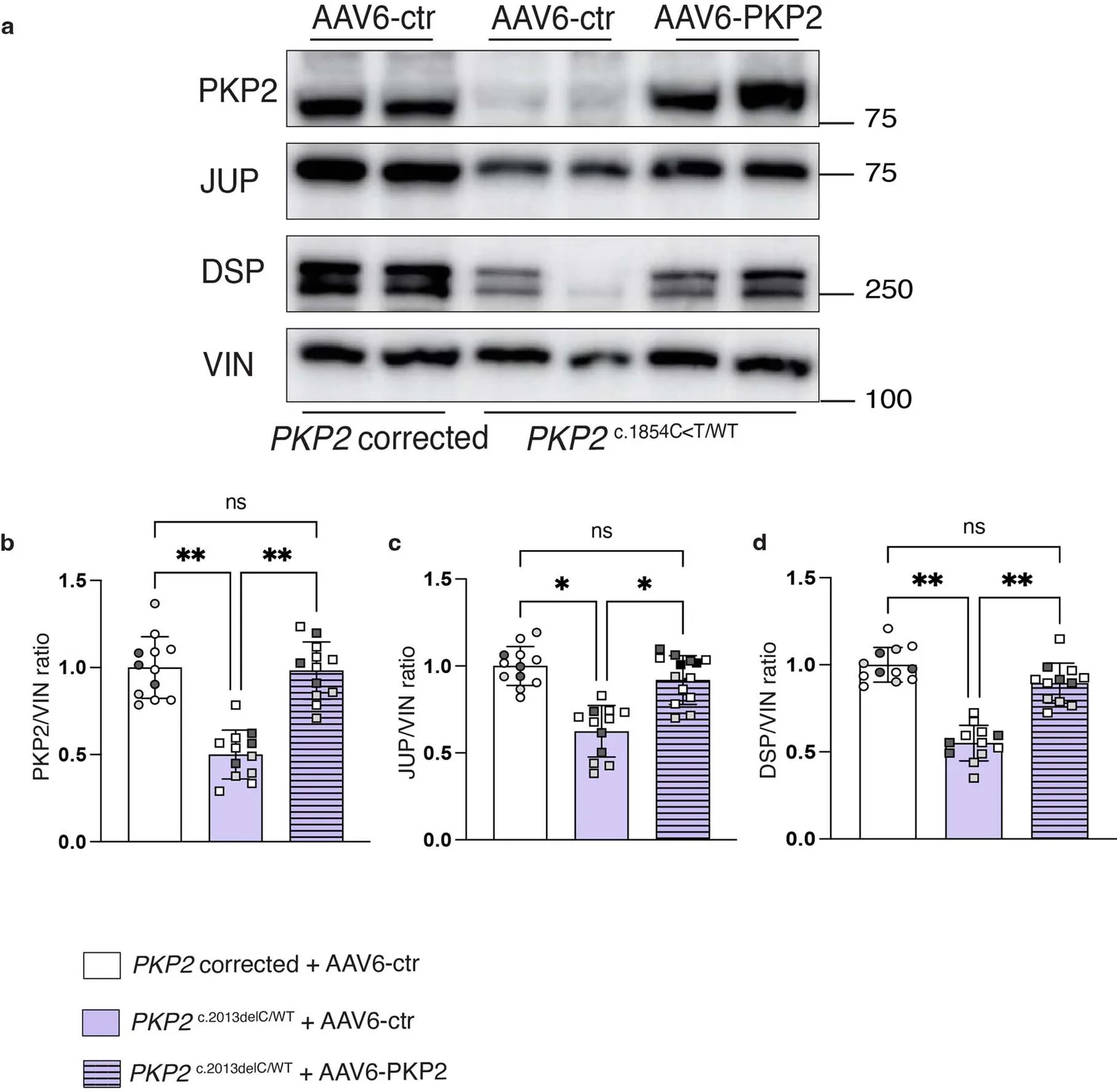
PKP2 restoration in iPS-cell-derived CMs harboring the pathogenic variant *PKP2* c.1845C < T, leads to a recovery of JUP and DSP protein levels. **a**, Representative immunoblots for PKP2, JUP and DSP in *PKP2*^c.1845C<T/WT^ iPS-cell CMs upon transduction with AAV6-ctr or AAV6-PKP2. VIN was used as a loading control. **b-d**, Quantification of (a), n = 3 biological replicates (distinct differentiations) and 6, 3 and 3 technical replicates per differentiation respectively. Data is presented as mean +/− s.e.m. Statistical significance is derived from biological replicates and is determined with one-way ANOVA (Tukey’s post-hoc test) with p-value at ****P < 0.0001, ***P < 0.001, **P < 0.01, *P < 0.05, and not significant (ns). *P*-values (*PKP2*^c.1845C<T/WT^+ AAV6-ctr vs *PKP2*^c.1845C<T/WT^+ AAV6-PKP2): (b) = 0.016, (c) = 0.0366, (d) = 0.0016. Source data

To start examining the effects of PKP2 restoration in a human-relevant cell model, we utilized a patient-derived iPS cell line, harboring the pathogenic mutation *PKP2* c.2013delC (*PKP2*^c.2013delC/WT^) and generated isogenic control cells as a reference^20^. To determine a suitable dosage, we delivered two different viral loads (v.l.) (v.l.1 = 0.5 × 10^3^ viral genomes (v.g.) per cell, v.l.2 = 5 × 10^3^ v.g. per cell) of an AAV6 vector expressing the wild-type human *PKP2* gene under the control of a cytomegalovirus (CMV) immediate-early promoter (AAV6–PKP2) (Fig. 1a). Molecular analysis of the transduced iPS-cell-derived CMs revealed a load-dependent response in PKP2 protein levels, while transduction with 5 × 10^3^ v.g. per cell resulted in complete restoration of the PKP2 protein levels in the mutant CMs (Fig. 1c,d). To assess the effect of PKP2 restoration on desmosomal integrity, we next transduced the *PKP2* mutant iPS-cell-derived CMs with either AAV6–PKP2 or an empty AAV6 vector (AAV6-ctr) and performed mRNA and protein analysis at 4 days and 7 days post-infection, respectively (Fig. 1b). As a reference, we also treated the *PKP2*-corrected iPS-cell-derived CMs with an equal titer of the AAV6-ctr construct. Real-time polymerase chain reaction (PCR) analysis did not show any effects of the exogenously delivered *PKP2* on the mRNA expression levels of other desmosomal components, including JUP, DSP, DSG2 and DSC2 (Extended Data Fig. 1a–e). In contrast, western blot analysis revealed that restoration of PKP2 protein levels induced subsequent recovery of the desmosomal proteins, JUP and DSP, in the AAV6–PKP2-treated *PKP2*^c.2013delC/WT^ iPS-cell-derived CMs, while the DSC2 and DSG2 protein levels remained unaffected (Fig. 1e–j). Performing these experiments in additional iPS-cell-derived CM lines harboring a different pathogenic *PKP2* mutation, *PKP2* c.1854C>T (*PKP2*^c.1854C>T/WT^) corroborated these findings, showing rescue of desmosomal protein content in response to viral delivery of human (h)*PKP2* (Extended Data Fig. 2a–d). Together, these results indicated that restoration of physiological levels of PKP2 protein improved desmosomal assembly in human CMs harboring *PKP2* truncating variants.

**Fig. 2:**
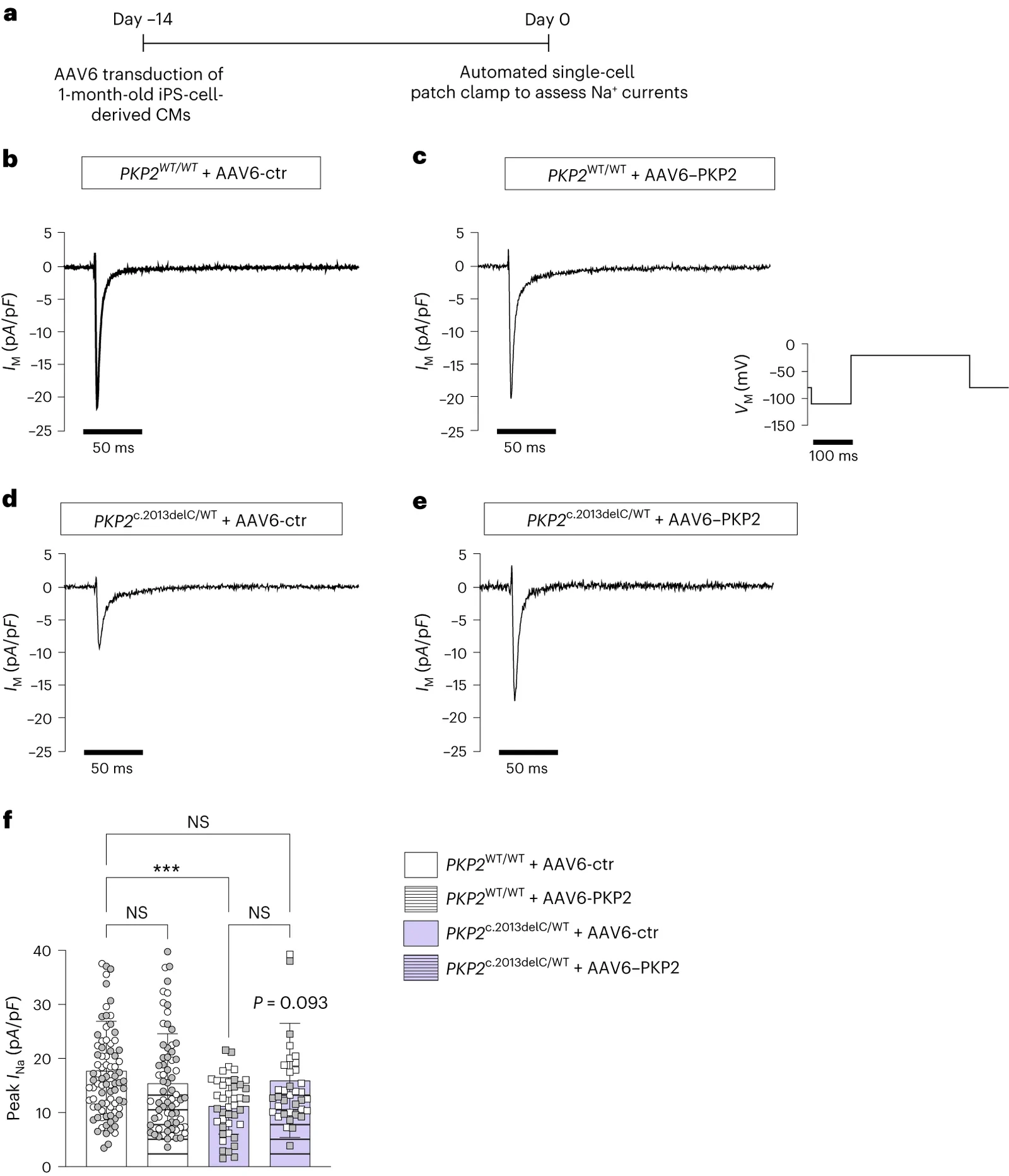
AAV-mediated restoration of PKP2 in *PKP2*^c.2013delC/WT^ mutant iPS-cell-derived CMs improves sodium conduction. **a**, Graphical representation of the experimental workflow. **b**–**e**, Representative voltage-gated sodium currents activated by voltage steps to between −80 mV and −20 mV from a holding potential of −110 mV. Experimental groups: *PKP2*^WT/WT^ + AAV6-ctr shown in **b**, *PKP2*^WT/WT^ + AAV6-PKP2 shown in **c**, *PKP2*^c.2013delC/WT^ + AAV6-ctr shown in **d**, *PKP2*^c.2013delC/WT^ + AAV6-PKP2 shown in **e**. **f**, Graph showing the peak transient *I*_Na_ density for each experimental group. Colored dots represent distinct CM differentiation. *PKP2*^WT/WT^ + AAV6-ctr: *n* = 39 single CMs (diff.1) + 48 single CMs (diff.2), *PKP2*^WT/WT^ + AAV6–PKP2: *n* = 26 single CMs (diff.1) + 44 single CMs (diff.2), *PKP2*^c.2013delC/WT^ + AAV6-ctr: *n* = 21 single CMs (diff.1) + 20 single CMs (diff.2), *PKP2*^c.2013delC/W^ + AAV6–PKP2: *n* = 23 single CMs (diff.1) + 14 single CMs (diff.2). Data are presented as mean values ± s.e.m. Statistical significance was estimated with one-way ANOVA, Tukey correction with *P* value at ****P < 0.0001, ***P < 0.001, **P < 0.01, *P < 0.05, and not significant (NS). *P* values (**f**): *PKP2*^WT/WT^ + AAV6-ctr versus *PKP2*^c.2013delC/WT^ + AAV6-ctr = 0.0008; *PKP2*^c.2013delC/WT^ + AAV6-ctr versus *PKP2*^c.2013delC/WT^ + AAV6–PKP2 = 0.0930; *PKP2*^WT/WT^ + AAV6-ctr versus *PKP2*^c.2013delC/WT^ + AAV6–PKP2 = 0.7177. Source data

As severe ventricular arrhythmia is the hallmark of ACM, we then assessed whether our 2D in vitro model recapitulates the arrhythmic substrate caused by ion channel irregularities reported to be present in other relevant studies^26,27^. We, therefore, conducted high-throughput automated patch clamp on iPS-cell-derived CMs to evaluate sodium currents (Fig. 2a). Our study included four distinct conditions, with each experiment conducted on two separate differentiations of CMs.

In comparing sodium conduction between the mutant *PKP2* CM line and the isogenic control line we were able to demonstrate a significant reduction in sodium conduction in the mutant CMs, thus successfully replicating the arrhythmic substrate characteristic of ACM (Fig. 2b,c). We subsequently sought to assess the potential of the AAV6–PKP2 gene replacement approach to ameliorate this sodium conduction impairment in the mutant CMs. Our data reveal that treatment with AAV6–PKP2 effectively diminished the differences in sodium conduction, restoring it to a level comparable to that of the isogenic control line (Fig. 2d–f).

These findings show that a gene replacement approach improves sodium conduction in mutant CMs, which might suggest it could rescue the arrhythmic phenotype in patients with ACM.

### PKP2 restoration enhances contractile function in engineered human myocardium

**Fig. 3:**
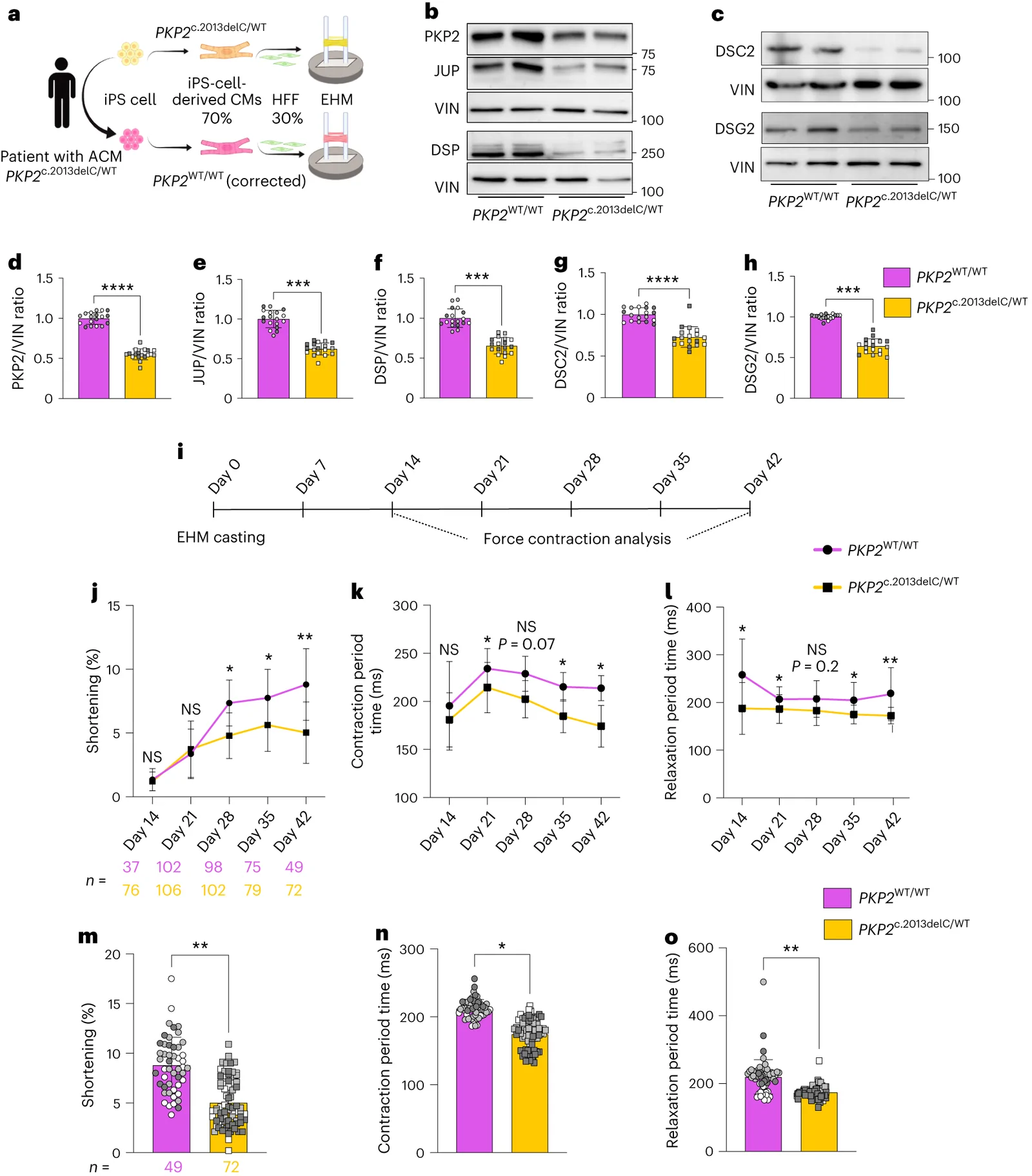
*PKP2*^c.2013delC/WT^ mutant EHM exhibit impaired contractile function compared to the isogenic control. **a**, Schematic overview of the generation of *PKP2* EHM. **b**, Representative immunoblot for the desmosomal proteins PKP2, JUP and DSP. VIN is used as a loading control. **c**, Representative immunoblot for the desmosomal proteins DSC2 and DSG2. VIN is used a loading control. **d**, Quantification of PKP2 protein levels. **e**, Quantification of JUP protein levels. **f**, Quantification of DSP protein levels. **g**, Quantification of DSC2 protein levels. **h**, Quantification of DSG2 protein levels. Colored dots represent distinct CM differentiations, *n* = 6 technical replicates and 3 biological replicates per condition. Data are presented as mean values ± s.e.m. Statistical significance is derived by biological replicates and determined by unpaired, two-tailed Student’s *t*-test, *P* value at ****P < 0.0001, ***P < 0.001, **P < 0.01, *P < 0.05, and not significant (NS). **i**, Timeline for assessing contractility of *PKP2*^WT/WT^ and *PKP2*^c.2013delC/WT^ EHM. **j**, Trendline showing % pole bending as a measure of force of contraction for *PKP2*^WT/WT^ and *PKP2*^c.2013delC/WT^ EHM under baseline conditions at different time points. **k**, Trendline showing contraction time (from 20% to 80% contraction in ms) for *PKP2*^WT/WT^ and *PKP2*^c.2013delC/WT^ EHM under baseline condiitons at different time points. **l**, Trendline showing relaxation time (from 20% to 80% relaxation in ms) for *PKP2*^WT/WT^ and *PKP2*^c.2013delC/WT^ EHM under baseline conditions at different time points. Statistics: one-way ANOVA with Tukey’s post-hoc test was performed between all groups at each time point, *n* = 3 biological replicates (exact number for technical replicates is indicated on the figure). **m**, Graph displaying force of contraction (as % pole bending) for *PKP2*^WT/WT^ and *PKP2*^c.2013delC/WT^ EHM on day 42 of maturation. **n**, Graph displaying contraction time (ms) for *PKP2*^WT/WT^ and *PKP2*^c.2013delC/WT^ EHM on day 42 of maturation. **o**, Graph displaying relaxation time (ms) for *PKP2*^WT/WT^ and *PKP2*^c.2013delC/WT^ EHM on day 42 of maturation. Colored dots represent distinct CM differentiations, *n* = 3 biological replicates (*PKP2*^WT/WT^ EHM: *n* = 17 (diff.1), 21 (diff.2) and 11 (diff.3) technical replicates. *PKP2*^c.2013delC//WT^ EHM: *n* = 19 (diff.1), 23 (diff.2) and 30 (diff.3) technical replicates). Data are presented as mean values ± s.e.m. Statistical significance is derived from biological replicates and determined by unpaired, two-tailed Student’s *t*-test, *P* value at ****P < 0.0001, ***P < 0.001, **P < 0.01, *P < 0.05, and not significant (NS). *P* values: 0.0002 (**d**), <0.0001 (**e**), 0.010 (**f**), <0.0001 (**g**), 0.0003 (**h**), 0.095 (42-day time point) (**j** and **m**), 0.0176 (42-day time point) (**k** and **n**) and 0.059 (42-day time point) (**l** and **o**). Source data

**Extended Data Fig. 3:**
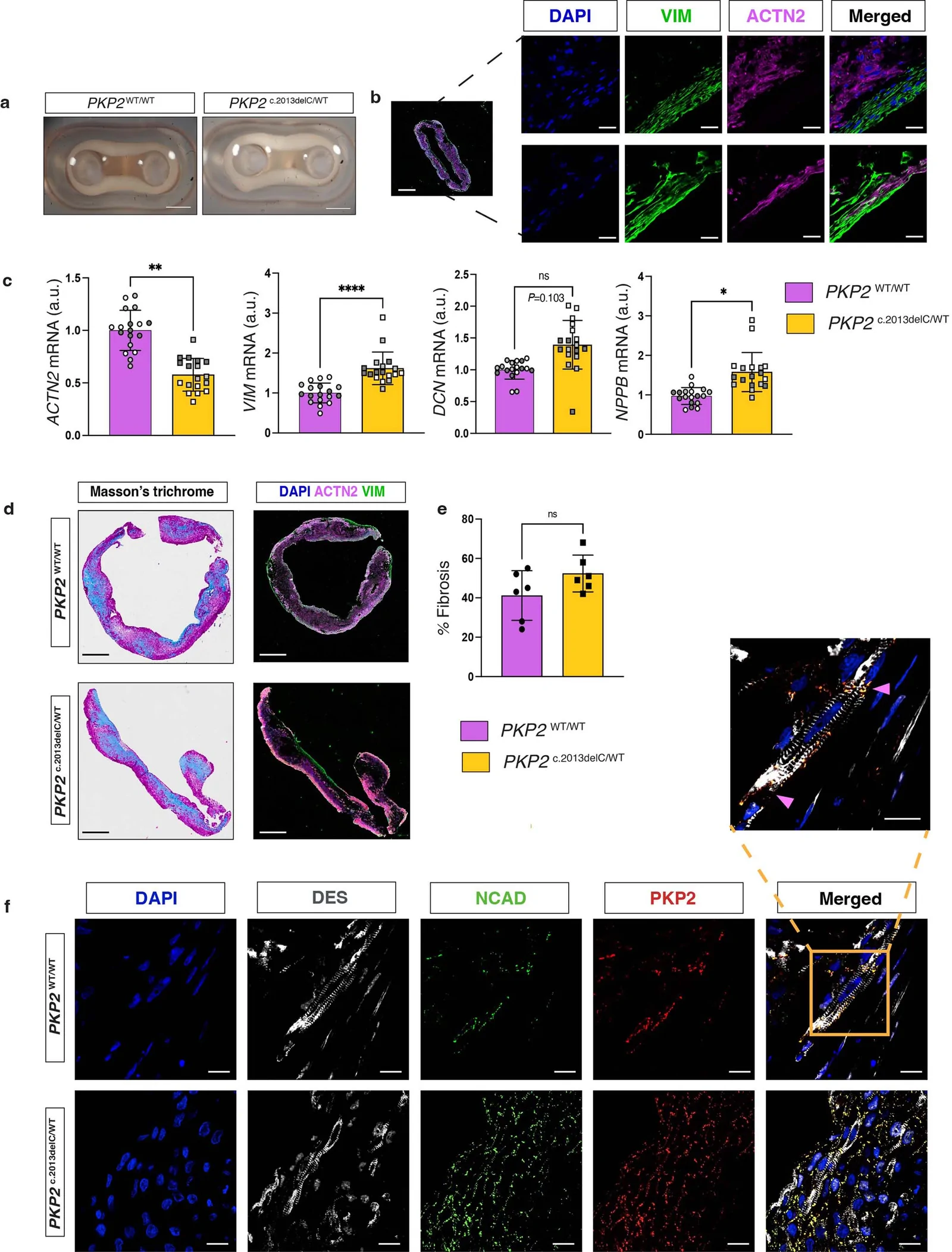
The *PKP2* mutant EHM exhibit similar structural and intercellular junction organization as its isogenic control. **a**, Representative brightfield images of 6-week-old mutant and isogenic control EHM under baseline. Scale bar is at 1mm. **b**, On the left side: representative overview immunofluorescent staining of a paraffin-embedded EHM section. Scale bar is at 500μm. On the right side: representative high magnification fluorescent images of the same EHM section to show cardiomyocyte and fibroblast regions within the tissue. DAPI = blue, VIM = magenta, ACTN2 = green. Scale bar is at 20μm. **c**, Gene expression of ACTN2, VIM, DCN and NPPB on the mutant tissues compared to the isogenic control. Data is normalized to the housekeeping gene *GUS*. Colored dots represent distinct CM differentiations, n = 6 technical replicates and 3 biological replicates per condition. Data is presented as means +/− s.e.m. Statistical significance is derived from biological replicates and is determined by unpaired, two-tailed Student t-test, p-value at *****P < 0.0001, ***P < 0.001, **P < 0.01, *P < 0.05, and not significant (ns). ACTN2: *P*-value = 0.0077, VIM: *P*-value < 0.0001, DCN: *P*-value = 0.1033, NPPB: *P*-value = 0.0401. **d***, On the left column:* representative images of a Masson’s trichrome staining on paraffin-embedded EHM. Collagen = blue, muscle = red and nuclei = brown. *On the right column:* representative immunofluorescent images from paraffin-embedded EHM. ACTN2 = magenta, VIM = green and DAPI = in blue. Scale bar is at 500 μm. **e**, Quantification of the Masson’s trichrome staining in (d). Each dot represents a mouse. Data is presented as means +/− s.e.m. Statistical significance is determined by unpaired, two-tailed Student t-test, p-value at ****P < 0.0001, ***P < 0.001, **P < 0.01, *P < 0.05, and not significant (ns). **f**, Representative immunofluorescent images displaying the intercalated disc (ID) structure on EHM. NCAD = green, PKP2 = red, DES = gray and DAPI = blue. Scale bar is at 20μm and at 10μm for the zoomed image on the top right. Pink arrowheads indicate a representative ID-like structure on a cardiomyocyte. This experiment has been performed three times independently with similar results. Source data

As PKP2 plays a major role in intercellular binding and interaction, we next generated three-dimensional (3D) EHM composed of 70% iPS-cell-derived CMs combined with 30% human foreskin fibroblasts (HFFs) in a collagen type I-based hydrogel (Fig. 3a and Extended Data Fig. 3a,b). This model provides a more mature CM phenotype compared to 2D cell cultures, while enabling the sequential assessment of contractile properties at various stages of tissue maturation^28^.

Interestingly, *PKP2*^c.2013delc/WT^ tissues exhibited a significant reduction in expression of cardiac markers, including α-actinin-2 (*ACTN2*) accompanied by an increase in the expression levels of several fibroblast markers such as vimentin (*VIM*) and decorin (*DCN*) compared to the isogenic control (Extended Data Fig. 3c). Furthermore, the mutant tissues showed significantly higher levels of the stress-related gene *NPPB* (Extended Data Fig. 3c). Notably we did not detect the presence of fibrofatty markers in either of the two genotypes. Despite these transcriptional differences, our histological examination using Masson’s trichrome staining did not reveal substantial differences in fibrosis between the two genotypes at the tissue level (Extended Data Fig. 3d,e). Immunofluorescence assays on 6-week-old EHM revealed colocalization of PKP2 with the ID-specific marker N-cadherin (NCAD), however a more organized ID-like structure was more evident in the control tissues compared to the mutant tissues (Extended Data Fig. 3f). These results suggest that both the *PKP2* mutant and isogenic control EHM develop a myocardium-like structure, albeit with differences in the organization of CM junctions, which appear to be improved in both lines compared to the 2D cultures.

**Extended Data Fig. 4:**
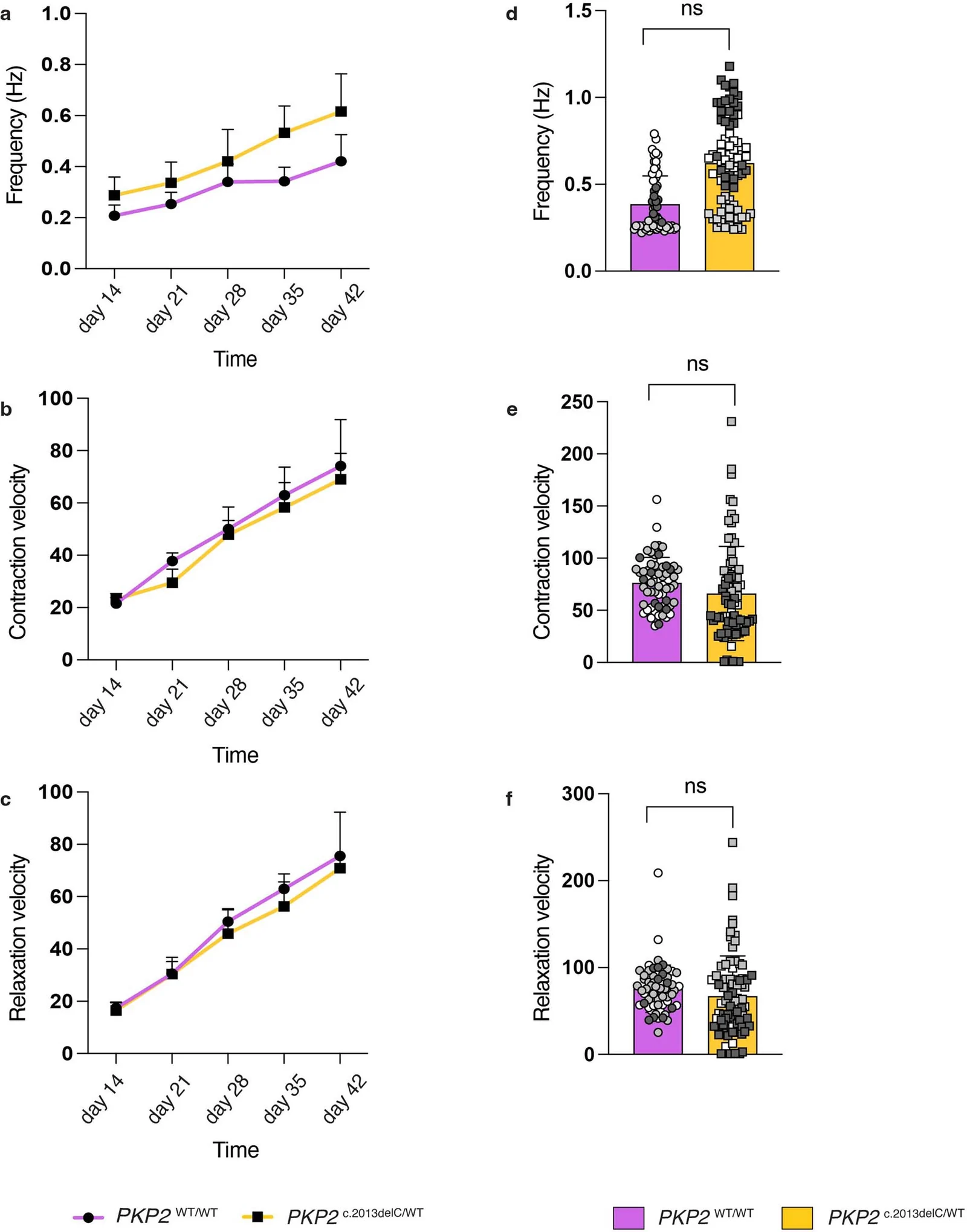
*PKP2*^c.2013delC/WT^ EHM do not exhibit an altered beating frequency, contraction velocity and relaxation velocity compared to control. **a-c**, Trendlines showing beating frequency (Hz), contraction velocity and relaxation velocity in *PKP2* ^WT/WT^ and *PKP2*^c.2013delC/WT^ EHM at different timepoints after transduction with either AAV6-PKP2 or AAV6-ct, n = 3 biological replicates (three distinct EHM batches). **d-f**, Graphs displaying beating frequency (Hz), contraction velocity and relaxation velocity in *PKP2* ^WT/WT^ and *PKP2*^c.2013delC/WT^ EHM after transduction with either AAV6-PKP2 or AAV6-ctr, on day 42 of maturation, n = 3 biological replicates (three distinct EHM batches), batch 1 = 17 isogenic control and 19 mutant tissues, batch 2 = 32 isogenic control and 27 mutant tissues and batch 3 = 11 isogenic control and 30 mutant tissues. Data is presented as means +/− s.e.m. Statistical significance is derived by biological replicates and is determined by unpaired, two-tailed Student t-test p-value at ****P < 0.0001, ***P < 0.001, **P < 0.01, *P < 0.05, and not significant (ns). Source data

Next, we sought to investigate the desmosomal composition of the mutant and isogenic control EHM. Molecular analysis by western blot confirmed a two-fold decline of PKP2 protein levels in *PKP2*^c.2013delC/WT^ EHM compared to *PKP2*^WT/WT^ EHM, accompanied by a reduction in JUP and DSP protein levels, which is consistent with the molecular phenotype observed in 2D cultures (Fig. 3b,d–f). The desmosomal cadherins DSC2 and DSG2 also showed reduced protein levels in the mutant tissues compared to the control tissues, which for DSC2 was not observed in our 2D model (Figs. 1i and 3c,g–h). This discrepancy might be attributed to the different levels of maturation that the two models present. It is also possible that the mechanical stress that is involved in EHM culture exacerbates the overall phenotype observed, therefore leading to a decline of DSC2 protein. In parallel, video-optical recordings of EHM contraction revealed a significant decline in contractile function (decrease in force of contraction) in *PKP2* mutant EHM compared to the *PKP2*^WT/WT^ EHM (Fig. 3i–o). Other contractility properties, including beating frequency, contraction velocity and relaxation velocity, did not show significant differences between mutant and isogenic control tissues throughout maturation (Extended Data Fig. 4a–g).

**Fig. 4:**
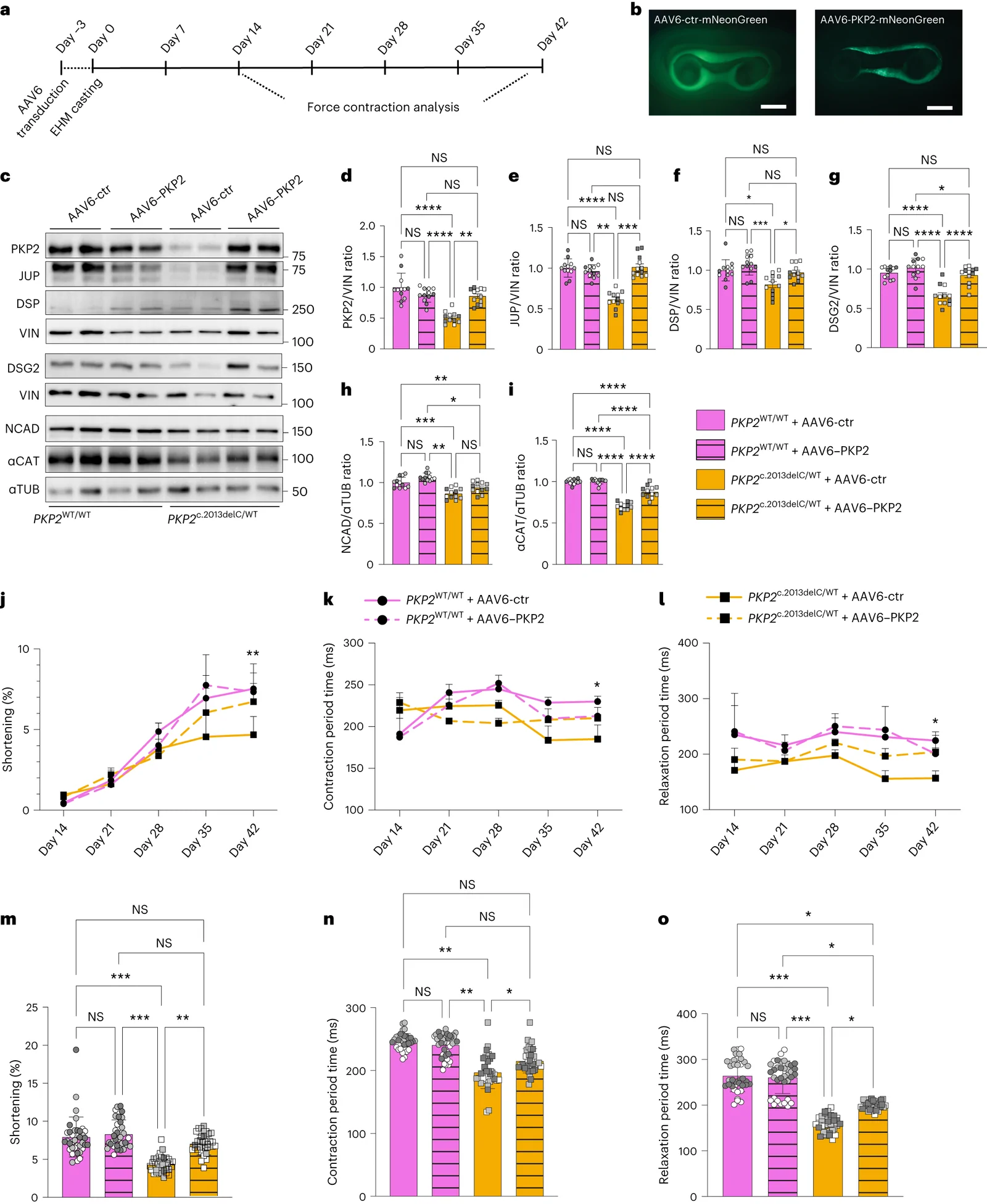
AAV-mediated restoration of PKP2 in *PKP2*^c.2013delC/WT^ EHM leads to an increase in desmosomal and junctional protein levels, which further translates into improved contractility. **a**, Timeline for the AAV transduction and maturation of *PKP2*^WT/WT^ and *PKP2*^c.2013delC/WT^ EHM with either AAV6-ctr or AAV6–PKP2. **b**, Representative fluorescent image of 6-week-old EHM transduced with either AAV6-ctr (left) or AAV6–PKP2 fused with the mNeongreen fluorescent protein (right). Scale bar, 1 mm. This experiment was repeated independently three times with similar results. **c**, Representative immunoblots for PKP2, JUP, DSP, DSG2, NCAD and αCAT in 6-week-old *PKP2*^WT/WT^ and *PKP2*^c.2013delC/WT^ EHM, transduced either with AAV6-ctr or AAV6–PKP2. VIN was used as a loading control for the desmosomal proteins, whereas αTUB was used for the quantification of NCAD and αCAT. **d**, Quantification of PKP2 protein levels. **e**, Quantification of JUP protein levels. **f**, Quantification of DSP protein levels. **g**, Quantification of DSG2 protein levels. **h**, Quantification of NCAD protein levels. **i**, Quantification of αCAT protein levels. Colored dots represent distinct CM differentiations, *n* = 6 technical replicates and 3 biological replicates per condition. Data are presented as mean values ± s.e.m. Statistical significance is derived from biological replicates and determined by one-way ANOVA (Tukey’s post-hoc test), *P* value at ****P < 0.0001, ***P < 0.001, **P < 0.01, *P < 0.05, and not significant (NS). **j**, Trendline showing % pole bending as a measure of force of contraction for *PKP2*^WT/WT^ and *PKP2*^c.2013delC/WT^ EHM after AAV6 transduction at different time points. **k**, Trendline showing contraction time (from 20% to 80% contraction in ms) for *PKP2*^WT/WT^ and *PKP2*^c.2013delC/WT^ EHM after AAV6 transduction at different time points. **l**, Trendline showing relaxation time (from 20% to 80% relaxation in ms) for *PKP2*^WT/WT^ and *PKP2*^c.2013delC/WT^ EHM after AAV6 transduction at different time points. Statistics: one-way ANOVA with Tukey’s post-hoc test was performed between all groups at each time point, *n* = 3 biological replicates and 36 technical replicates per condition (12 tissues per CM differentiation). **m**, Graph summarizing force of contraction (% pole bending) for *PKP2*^WT/WT^ and *PKP2*^c.2013delC/WT^ EHM on day 42 of maturation. **n**, Graph summarizing contraction time (ms) for *PKP2*^WT/WT^ and *PKP2*^c.2013delC/WT^ EHM on day 42 of maturation. **o**, Graph summarizing relaxation time (ms) for *PKP*2^WT/WT^and *PKP2*^c.2013delC/WT^ EHM on day 42 of maturation, *n* = 3 biological replicates and 36 technical replicates (12 tissues per condition). Data are presented as mean values ± s.e.m. Statistical significance is derived from biological replicates and determined by one-way ANOVA (Tukey’s post-hoc test), *P* value at ****P < 0.0001, ***P < 0.001, **P < 0.01, *P < 0.05, and not significant (NS). *P* value (*PKP2*^c.2013delC/WT^ + AAV6-ctr versus *PKP2*^c.2013delC/WT^ + AAV6–PKP2): 0.0010 (**d**), 0.0006 (**e**), 0.3638 (**f**), <0.0001 (**g**), 0.3156 (**h**), <0.0001 (**i**), 0.0033 (**j** and **m**), 0.0274 (**k** and **n**) and 0.0344 (**l** and **o**). Source data

**Extended Data Fig. 5:**
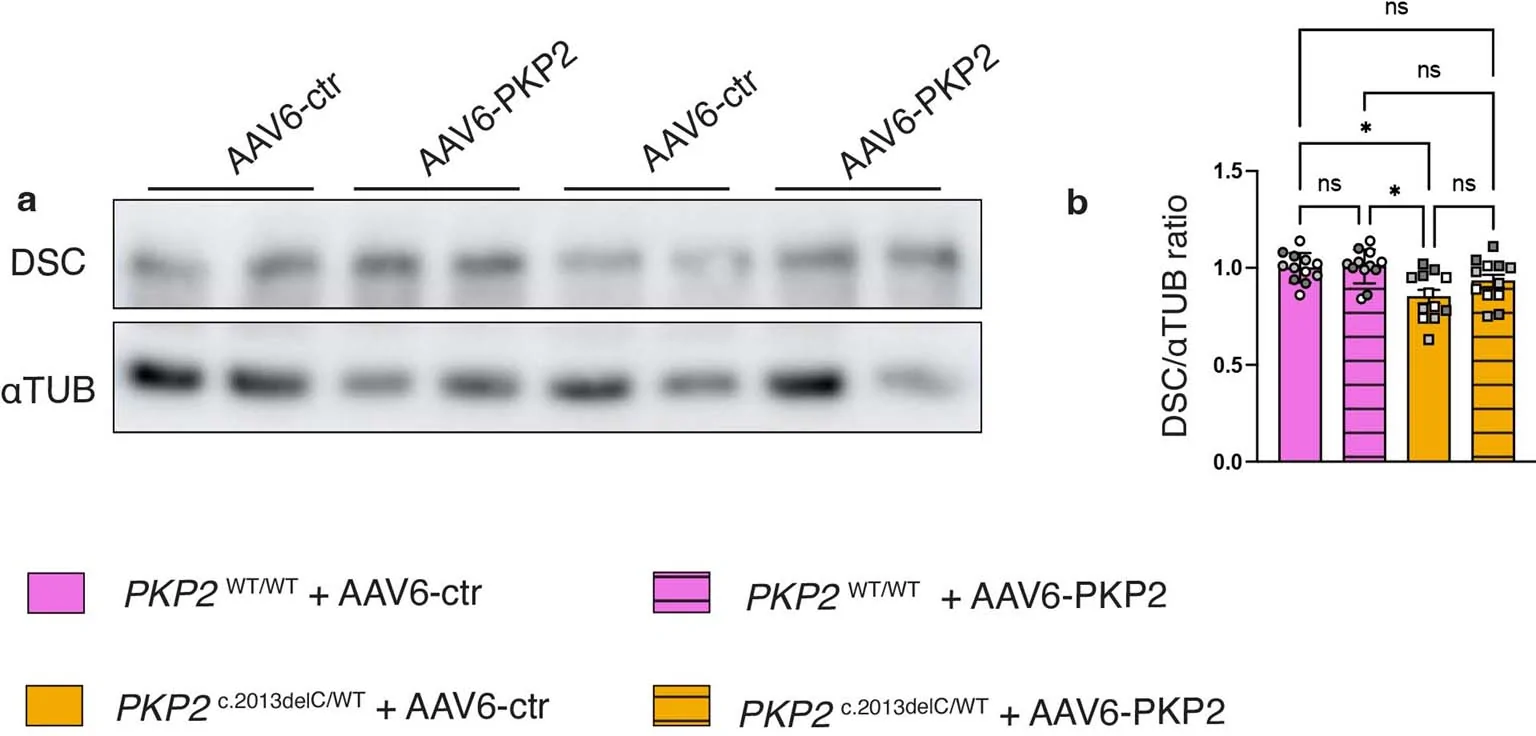
AAV-PKP2 treatment does not alter desmocollin levels in *PKP2*^c.2013delC/WT^ EHM. **a**, Representative immunoblots for DSC2 on PKP2 mutant and isogenic control EHM upon transduction with either AAV-ctr or AAV-PKP2. **b**, Quantification of (a). Colored dots represent distinct batches of differentiation, n = 4 technical replicates and 3 biological replicates. Data is presented as means +/− s.e.m. Statistical significance is derived by biological replicates and determined by one-way ANOVA (Tukey’s post-hoc test) with p-value at ****P < 0.0001, ***P < 0.001, **P < 0.01, *P < 0.05, and not significant (ns). *P*-value = 0.0821. Source data

To determine whether PKP2 restoration could improve the functional phenotype observed in mutant tissues, we transduced *PKP2*^c.2013delC/WT^ and *PKP2*^WT/WT^ iPS-cell-derived CMs with either the AAV6–PKP2 or the AAV6-ctr and reconstituted the CMs to EHM 3 days post-transduction (Fig. 4a,b). Our results showed successful PKP2 restoration in the *PKP2*^c.2013delC/WT^–AAV6–PKP2 tissues at 6 weeks after EHM formulation, which was paralleled by a strong increase in JUP, DSP and DSG2 protein levels (Fig. 4c–g). Interestingly, DSC2 protein levels did not respond to PKP2 restoration (Extended Data Fig. 5a,b). Apart from the desmosomal proteins, we also assessed the levels of other ID-related factors including NCAD and αCAT. PKP2 restoration was sufficient to restore the levels of these proteins in the mutant tissues, potentially implying that a stronger junction is formed between the adjacent CMs (Fig. 4c,h,i). Importantly, *PKP2* overexpression in the *PKP2*^WT/WT^ tissues did not lead to a significant increase in PKP2 or other desmosomal protein levels, potentially implying that an excess of PKP2 is being degraded by the cellular housekeeping machinery.

At a functional level, AAV6–PKP2 transduction resulted in a progressive improvement in contraction amplitude and normalization of the altered contraction kinetics, that is, an elongation of contraction and relaxation duration towards *PKP2*^WT/WT^ levels in the *PKP2*^c.2013delC/WT^ EHM from day 28, reaching statistical significance on day 42 post-casting (Fig. 4j–o). Noteworthily, *PKP2* expression in the AAV6–PKP2-treated isogenic control EHM did not lead to any side effects on the contractile phenotype, further supporting our hypothesis that *PKP2* overexpression is not detrimental to healthy CMs.

These data show that AAV-mediated restoration of PKP2 leads to molecular and functional benefits in ACM–EHM models with PKP2 haploinsufficiency.

### In vivo PKP2 delivery restores CM junctions

**Fig. 5:**
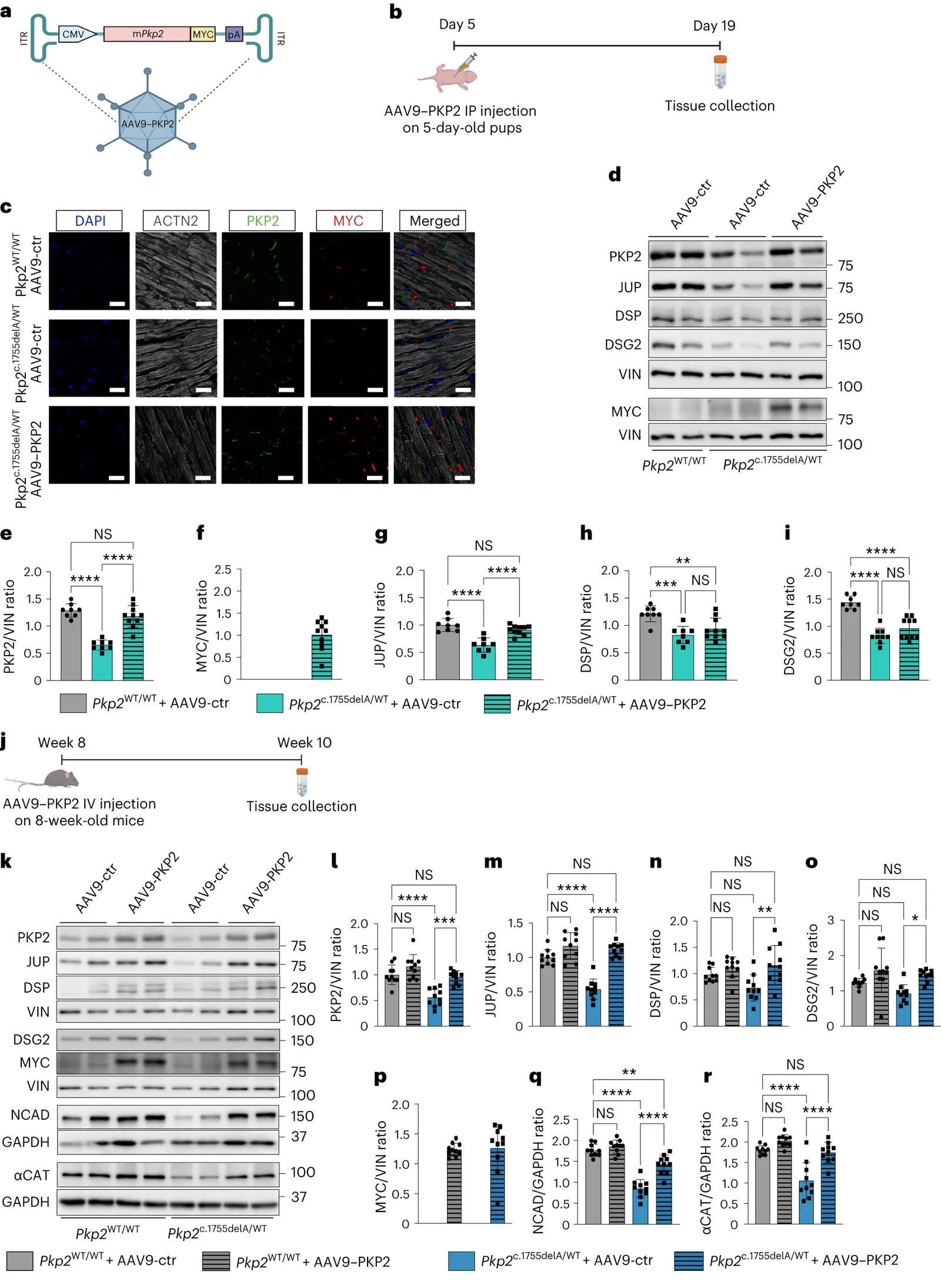
AAV-mediated restoration of PKP2 in *Pkp2*^c.1755delA/WT^ pups and adult mice leads to the recovery of desmosomal and non-desmosomal components of the ID. **a**, Graphical representation of the AAV expression cassette used in mice, ITR=Inverted Terminal Repeat Sequences. **b**, Workflow followed after the intraperitoneal (IP) AAV9–PKP2 administration in *Pkp2*^c.1755delA/WT^ and wild-type pups. **c**, Immunofluorescence on paraffin sections of mouse cardiac tissue showing localization of the exogenously introduced PKP2 in the injected hearts. DAPI, blue; ACTN2, gray; PKP2, green; MYC epitope, red; exogenous PKP2, yellow in merged image. Scale bar, 10 μm. This experiment was repeated independently 18 times (in 18 distinct mouse hearts) with the same results. **d**, Representative immunoblots for PKP2, MYC, JUP, DSP and DSG2 in *Pkp2*^WT/WT^ and *Pkp2*^c.1755delA/WT^ pups injected with either AAV9-ctr or AAV9–PKP2. VIN is used as a loading control. **e**, Quantification of PKP2 protein levels. **f**, Quantification of MYC protein levels. **g**, Quantification of JUP protein levels. **h**, Quantification of DSP protein levels. **i**, Quantification of DSG2 protein levels. Experimental groups: *Pkp2*^WT/WT^ pups injected with AAV9-ctr *n* = 8, *Pkp2*^c.1755delA/WT^ pups injected with AAV9-ctr *n* = 8, *Pkp2*^c.1755delA/WT^ pups injected with AAV9–PKP2 *n* = 10. **j**, Workflow followed after the intravenous (IV) AAV9–PKP2 administration in *Pkp2*^c.1755delA/WT^ and wild-type adult mice. **k**, Representative immunoblots for the desmosomal PKP2, MYC, JUP, DSP and DSG2 and NCAD and αCAT in *Pkp2*^WT/WT^ and *Pkp2*^c.1755delA/WT^ mice injected with either AAV9-ctr or AAV9–PKP2. VIN is used as a loading control for the desmosomal proteins, whereas GAPDH is used for the quantification of NCAD and αCAT. **l**, Quantification of PKP2 protein levels. **m**, Quantification of JUP protein levels. **n**, Quantification of DSP protein levels. **o**, Quantification of DSG2 protein levels. **p**, Quantification of MYC protein levels. **q**, Quantification of NCAD protein levels. **r**, Quantification of αCAT protein levels. Experimental groups: *Pkp2*^WT/WT^ mice injected with AAV9-ctr *n* = 10, *Pkp2*^WT/WT^ mice injected with AAV9–PKP2 *n* = 10, *Pkp2*^c.1755delA/WT^ mice injected with AAV9-ctr *n* = 10, *Pkp2*^c.1755delA/WT^ mice injected with AAV9–PKP2 *n* = 10. Data are presented as mean values ± s.e.m. Statistical significance is determined by one-way ANOVA (Tukey’s post-hoc test), *P* value at ****P < 0.0001, ***P < 0.001, **P < 0.01, *P < 0.05, and not significant (NS). *P* value (*Pkp2*^c.1755delA/WT^ + AAV9-ctr versus *Pkp2*^c.1755delA/WT^ + AAV9-PKP2): <0.0001 (**e**), <0.0001 (**g**), 0.3461 (**h**), 0.1607 (**i**), 0.002 (**l**), <0.0001 (**m**), 0.029 (**n**), 0.0122 (**o**), <0.0001 (**q**) and <0.0001 (**r**). Source data

To further increase clinical relevance, we made use of our previously described murine ACM model, harboring the mouse equivalent of the human pathogenic *PKP2* c.2013delC variant (*Pkp2*^c.1755delA/WT^). In brief, the *Pkp2*^c.1755delA/WT^ mice exhibit a significant reduction of cardiac desmosomal and adherens junction protein levels compared to *Pkp2*^WT/WT^ mice^20^. To administer *Pkp2* in vivo, we generated an AAV9 vector overexpressing the murine wild-type *Pkp2* fused to a MYC epitope to enable detection of the exogenously delivered PKP2 (AAV9–PKP2) (Fig. 5a). As a proof of concept, we initially administered an AAV9–PKP2 dose of 2 × 10^14^ v.g. kg^−1^ via intraperitoneal injections (IP) on 5-day-old *Pkp2* mutant pups and their wild-type control, and collected tissues 2 weeks after injection (Fig. 5b). Efficiency of transduction was approximately 76% of CMs (Supplementary Fig. 6a,b). Immunohistochemistry on paraffin sections of the murine hearts revealed correct localization of the exogenous PKP2 within the IDs, indicating functionality of the exogenous protein (Fig. 5c). Immunoblot analysis showed successful restoration of PKP2 protein levels in the treated mutant mice, corresponding to a significant recovery of JUP and a partial recovery in DSP and DSG2 (Fig. 5d–i), whereas DSC2 was not responsive to different PKP2 levels (Extended Data Fig. 7a, b). These findings are in line with what has been observed in our EHM models, consistently indicating the re-building of the desmosomal complex upon PKP2 restoration in our preclinical ACM models.

Since ACM symptoms often become apparent during early adulthood, we investigated the effect of AAV9–PKP2 administration at a more clinically relevant stage. For this purpose, we intravenously injected *Pkp2* mutant and wild-type mice at 2 months of age with a dose of 5 × 10^13^ v.g. kg^−1^ of either AAV9–PKP2 or AAV9-ctr (efficiency of transduction: 71% CMs in the left ventricle (LV)). Two weeks later we isolated tissues for molecular analysis (Fig. 5j). PKP2 restoration in the mutant mice resulted in elevated levels of the desmosomal proteins, JUP, DSP and DSG2, whereas DSC2 did not show an increase (Fig. 5k,l–o and Extended Data Fig. 7c,d). Importantly, PKP2 restoration also led to a significant recovery of adherens junction proteins, including NCAD and α-CAT, in line with what we observed on the EHM models (Fig. 4c,h,i). These results point to the formation of stronger junctions in the CMs of *Pkp2* mutant mice injected with AAV9–PKP2 and further support the therapeutic potential of PKP2 restoration in patients with ACM.

### PKP2 restoration improves cardiac function in mice

**Fig. 6:**
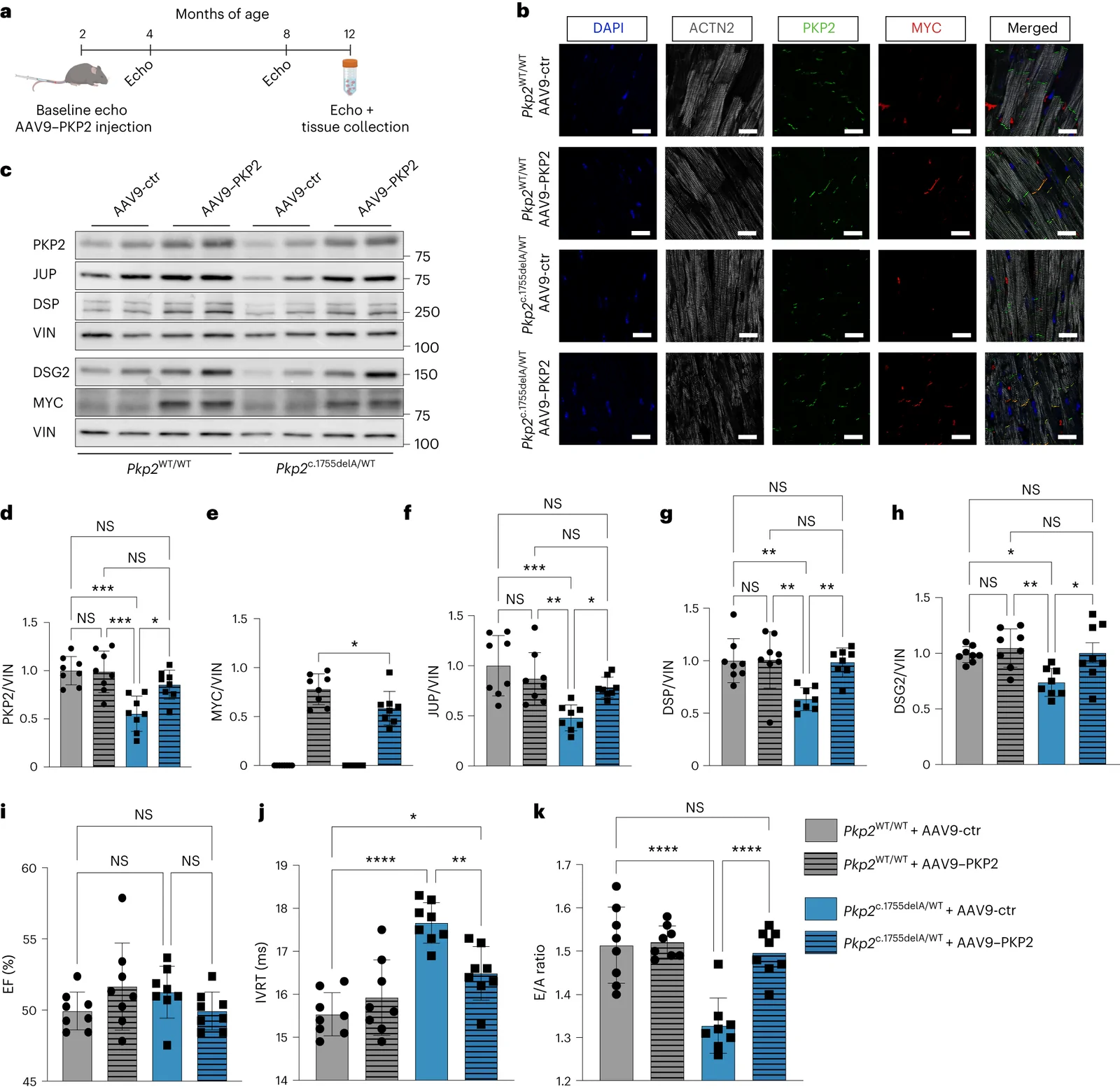
A single dose of AAV9-PKP2 in 8-week-old *Pkp2*^c.1755delA/WT^ mice prevents cardiac dysfunction at 12 months of age. **a**, Timeline for intravenous AAV9–PKP2 administration in *Pkp2*^c.1755delA/WT^ and long-term monitoring of cardiac function in adult mice. **b**, Immunofluorescence on paraffin sections of mouse cardiac tissue showing localization of the exogenously introduced PKP2 in the injected hearts. DAPI, blue; ACTN2, gray; PKP2, green; MYC epitope, red; exogenous PKP2, yellow in merged image. Scale bar, 10 μm. This experiment was performed independently ten times (in ten distinct mouse hearts) with the same results. **c**, Representative immunoblots for PKP2, MYC, JUP, DSP and DSG2 in *Pkp2*^WT/WT^ and *Pkp2*^c.1755delA/WT^ mice injected with either AAV9-ctr or AAV9–PKP2. VIN is used as a loading control. **d**, Quantification of PKP2 protein levels. **e**, Quantification of MYC protein levels. **f**, Quantification of JUP protein levels. **g**, Quantification of DSP protein levels. **h**, Quantification of DSG2 protein levels. Experimental groups: *Pkp2*^WT/WT^ mice injected with AAV9-ctr *n* = 8, *Pkp2*^WT/WT^ mice injected with AAV9–PKP2 *n* = 8, *Pkp2*^c.1755delA/WT^ mice injected with AAV9-ctr *n* = 8, *Pkp2*^c.1755delA/WT^ mice injected with AAV9–PKP2 *n* = 8. **i**, Graph showing EF in *Pkp2*^WT/WT^ and *Pkp2*^c.1755delA/WT^ mice injected with either AAV9-ctr or AAV9-PKP2. **j**, Graph showing IVRT in *Pkp2*^WT/WT^ and *Pkp2*^c.1755delA/WT^ mice injected with either AAV9-ctr or AAV9-PKP2. **k**, Graph showing E/A ratio in *Pkp2*^WT/WT^ and *Pkp2*^c.1755delA/WT^ mice injected with either AAV9-ctr or AAV9–PKP2. These measurements correspond to 12-month-old mice. Data are presented as mean values ± s.e.m. Statistical significance is determined by one-way ANOVA (Tukey’s post-hoc test), *P* value at ****P < 0.0001, ***P < 0.001, **P < 0.01, *P < 0.05, and not significant (NS). *P* value (*Pkp2*^c.1755delA/WT^ + AAV9-ctr versus *Pkp2*^c.1755delA/WT^ + AAV9-PKP2): 0.0101 (**d**), 0.0394 (**f**), 0.0052 (**g**), 0.0134 (**h**), 0.5622 (**i**), 0.0053 (**j**) and <0.0001 (**k**). Source data

**Extended Data Fig. 6:**
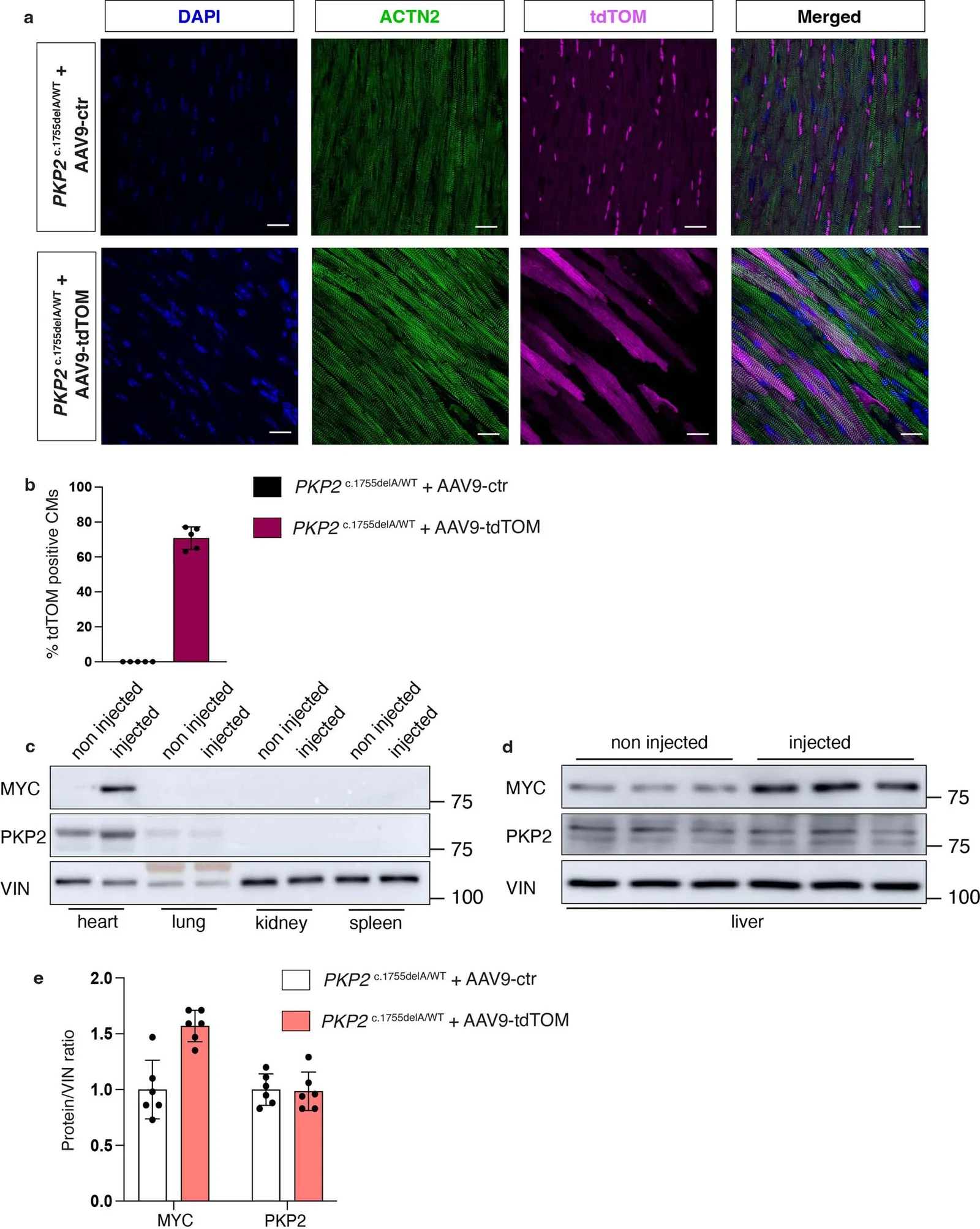
AAV9 transduces cardiomyocytes *in vivo* with high efficiency and with minor ectopic expression. **a**, Immunofluorescence on cardiac sections of mice transduced with AAV9-tdTOM to facilitate transduction efficiency assessment. Nuclei = DAPI, ACTN2 = green, tdTOM = magenta. Scale bar is at 20μm. **b**, Quantification of (a), n = 5 hearts, 10 regions per heart were assessed. **c-d**, Immunoblot for MYC and PKP2 on protein lysates of different mouse tissues. VIN was used as a loading control. **e**, Quantification of (d), n = 5 mice per condition. Data is presented as means +/− s.e.m. Source data

**Extended Data Fig. 7:**
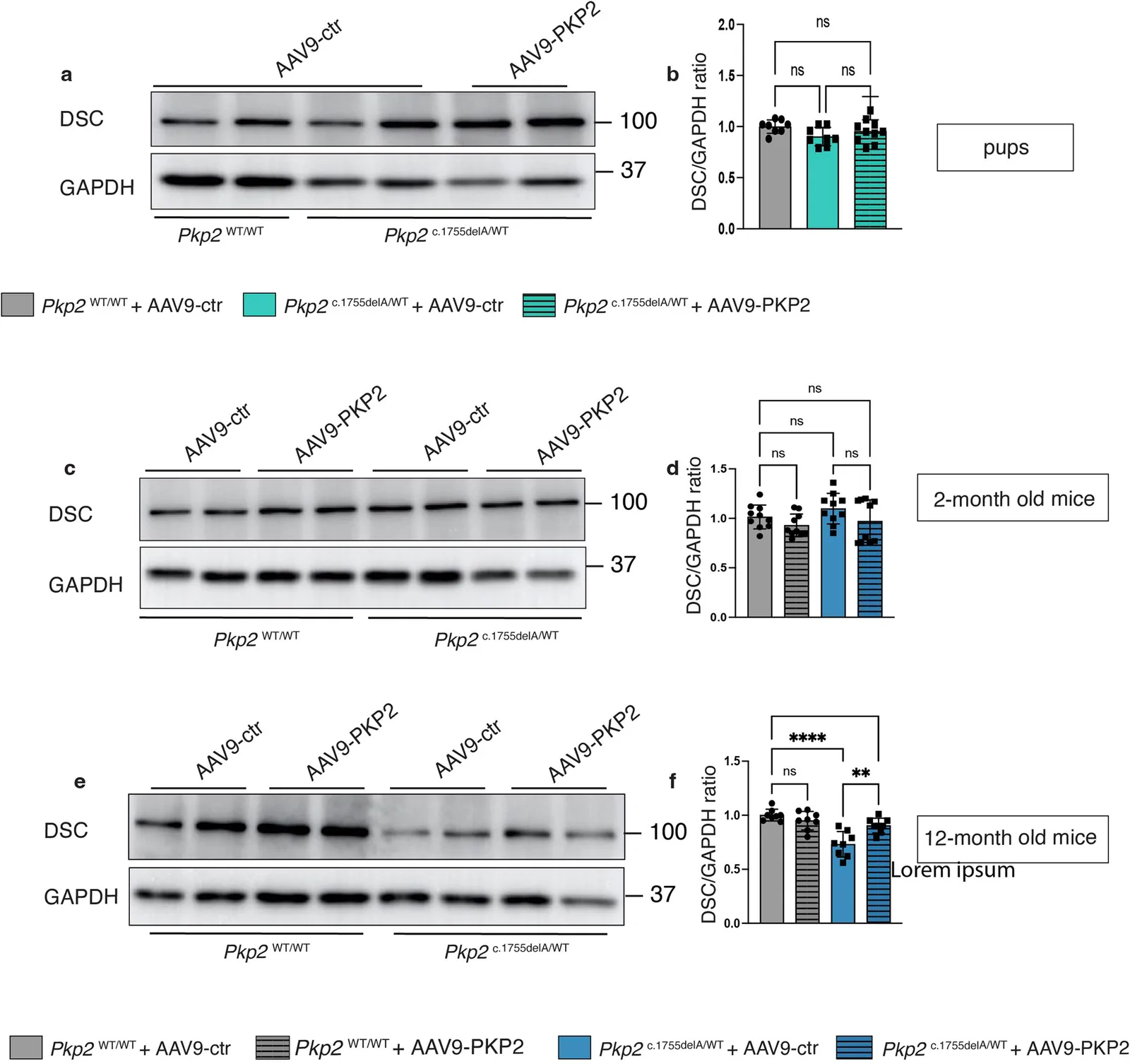
DSC2 is not consistently responsive to PKP2 restoration in the Pkp2^c.1755delA/WT^ murine models. **a, c, e**, Representative immunoblots for DSC2 in pups (n = 8,8 and 10 mice per group), 2-month-old (n = 10 mice per group) and 12-month-old mice (n = 8 mice per group) treated with AAV9-ctr or AAV9-PKP2. **b, d, f**, Quantification of each of the blots respectively. GAPDH has been used as a loading control for the quantification of the blots. Data is presented as mean +/− s.e.m. Statistical significance is determined by one-way ANOVA (Tukey’s post-hoc test) with p-value at ****P < 0.0001, ***P < 0.001, **P < 0.01, *P < 0.05, and not significant (ns). *P*-value (f) = 0.0017. Source data

**Extended Data Fig. 8:**
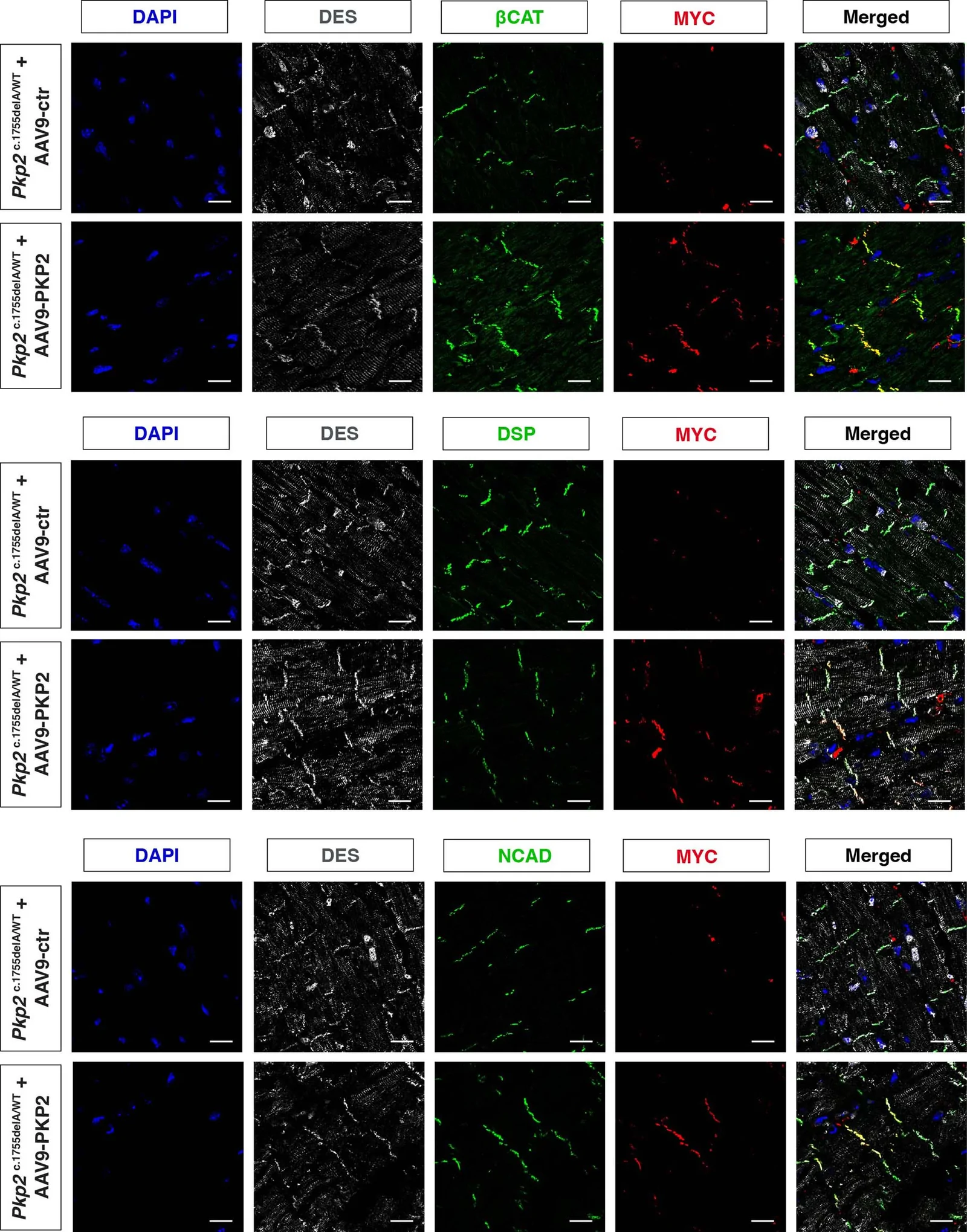
The exogenously delivered PKP2 specifically localizes at the ID of cardiomyocytes together with more junctional proteins. Representative immunofluorescent images of the exogenous-specific marker MYC colocalizing βCAT (top row), DSP (middle row) and NCAD (bottom row). Scale bar is at 20μm. This experiment has been repeated independently 10 times (in 10 distinct mouse hearts) with similar results.

**Extended Data Fig. 9:**
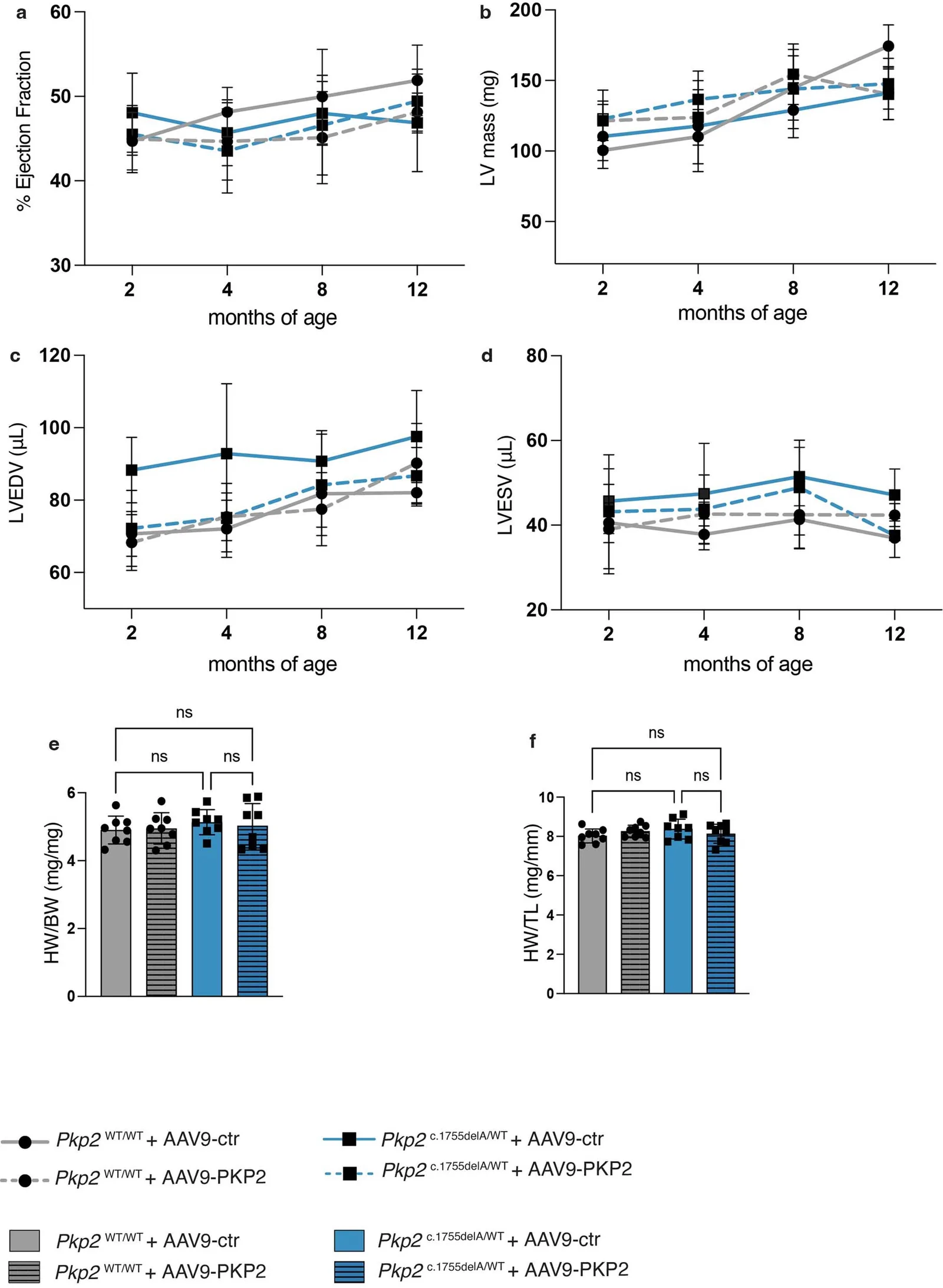
AAV-mediated overexpression of *Pkp2* does not cause an overt adverse cardiac phenotype in *Pkp2*^WT/WT^ 12-month-old mice. **a-d**, Trendlines showing % ejection fraction, left ventricle mass (LV mass), left ventricle end diastolic volume (LVEDV), left ventricle end systolic volume (LVESV) and in *Pkp2* ^WT/WT^ and *Pkp2*^c.1755delA/WT^ mice treated with either AAV9-PKP2 or AAV9-ctr at baseline (2 months old), 2 months (4 months old), 6 months (8 months old) and 10 months (12 months old) post injection, n = 8 mice per condition. **e**, Heart weight (HW) to body weight (BW) ratio in *Pkp*2^WT/WT^ and *Pkp2*^c.1755delA/WT^ mice treated with either AAV9-PKP2 or AAV9-ctr, n = 8 mice per group. **f**, Heart weight to tibia length (TL) ratio in *Pkp*2^WT/WT^ and *Pkp2*^c.1755delA/WT^ mice treated with either AAV9-PKP2 or AAV9-ctr, n = 8 mice per group. Data is presented as means +/− s.e.m. Statistical significance is determined by one-way ANOVA (Tukey’s post-hoc test), p-value at ****p < 0.0001, ***p < 0.001, **p < 0.01, *p < 0.05, and not significant (ns). Source data

To assess the consequences of long-term *Pkp2* administration on cardiac function in vivo, we injected both wild-type and mutant 2-month-old mice with a single dose (3 × 10^13^ v.g. kg^−1^) of either AAV9-ctr or AAV9–PKP2 and monitored heart function at 4, 8 and 12 months of age (Fig. 6a). We aged the mice up to 12 months as the mutant mice show progressive cardiac dysfunction with age, showing a significant decrease in the ratio of the early (E) to late (A) ventricular filling velocities (E/A ratio) and a significant increase in isovolumic relaxation time (IVRT) compared to the wild-type mice^20^. Ten months after a single injection, immunohistochemistry indicated the presence of exogenous PKP2 at the IDs of 12-month-old mouse hearts (Fig. 6b and Extended Data Fig. 8). Western blot analysis of the hearts extracted from AAV9–PKP2-treated mutant mice demonstrated restoration of the PKP2 protein levels and a significant recovery of the desmosomal proteins JUP, DSP and DSG2 including also DSC2 which was not responsive in the more immature models (Fig. 6c–h and Extended Data Fig. 7e,f). Interestingly, in line with our observations in the *PKP2*^WT/WT^ EHM, wild-type mice injected with the AAV9–PKP2 virus did not show a significant increase in PKP2 levels and also the protein levels of the assessed desmosomal components remained unaltered. In accordance with our previous study, echocardiographic analysis at baseline, 4 months, 8 months and 12 months of age did not reveal any significant differences in ejection fraction (EF) (Fig. 6i), LV mass, LV end diastolic volume (LVEDV) and LV end systolic volume (LVESV) between mutant and wild type (Extended Data Fig. 9a–e). Long-term exposure to either AAV-ctrl or AAV–PKP2 did also not influence these cardiac functional and morphological measures, further supporting that *Pkp2* overexpression does not impair cardiac physiology (Extended Data Fig. 9a–e). Importantly, assessment of E/A ratio and IVRT at the 12-month time point revealed a significant improvement in the AAV9–PKP2-treated mutant mice compared to the mutant mice injected with the AAV9-ctr (Fig. 6j,k). These data suggest that *Pkp2* administration in the mutant mice reinstates physiological gene expression profiles, improves electrical and mechanical coupling and eventually restores cardiac function. Morphological evaluation did not demonstrate significant differences in heart weight/body weight and heart weight/tibia length ratio among the different experimental groups (Extended Data Fig. 9e,f). Moreover, protein analysis of tissues that are prone to receive AAV9–PKP2 particles including lung, liver, spleen and kidney showed moderate expression of the exogenous PKP2 within the liver, which could be explained by the high liver tissue tropism of AAVs^29^ (Extended Data Fig. 6c–e). Overall, these data indicate that a single dose of AAV9–PKP2 is able to rescue the molecular and functional phenotype observed in the *Pkp2*^c.1755delA/WT^ adult mice without causing overt adverse effects in the heart and other organs.

## Discussion

PKP2 haploinsufficiency is often the underlying cause for ACM^13^. The data presented in this manuscript underscore the therapeutic potential of *PKP2* gene replacement therapy by showing both molecular and functional rescue in human-relevant preclinical in vitro and in vivo models of ACM, with no apparent detrimental effects of *PKP2* overexpression under healthy conditions. These are important findings that support the exploration of *PKP2* gene therapy as a targeted therapeutic treatment option for patients suffering from ACM.

Our data show that *PKP2* replacement under conditions of PKP2 haploinsufficiency can restore desmosomal integrity and CM function. These results are in line with a prior study by Inoue et al. that demonstrated AAV2-mediated PKP2 restoration in PKP2-deficient iPS-cell-derived CMs to restore other desmosomal components and enhance contractility^30^. By treating *PKP2*^c.2013delC/WT^ EHM with AAV6–PKP2 we were also able to show that the functional improvement is longlasting and progressive since the treated tissues showed constant improvement in contractile function from day 28 until day 42 post casting. Moreover, *PKP2* overexpression under healthy conditions did not appear to influence desmosomal integrity or CM function, which would be an important safety parameter for moving this technology into patients.

Next to our in vivo efficacy data, we provide evidence that a single systemic administration of AAV9–PKP2 in heterozygous mice harboring a pathogenic *PKP2* variant leads to restoration of desmosomal assembly in the transduced CMs, which is accompanied by the recovery of intercellular junction proteins NCAD and α-CAT. In time, *PKP2* gene therapy also prevents diastolic dysfunction in treated mutant mice at 12 months of age, while showing no effect in healthy wild-type littermates. This might be due to the lack of excessive PKP2 levels in both our wild-type in vitro and in vivo models. The lack of overexpression under healthy conditions might suggest the presence of a cellular compensatory mechanism correcting for an overdose of PKP2 protein. Similar observations have been reported for the exogenous expression of sarcomere genes in CMs. It has been demonstrated that viral expression of the hypertrophic cardiomyopathy-associated sarcomere gene myosin binding protein C (*MYBPC3*), can lead to the replacement of the endogenous protein without overexpression. This is due to UPS-mediated degradation of the excess amount of sarcomere proteins to preserve the stoichiometry of the sarcomere complex^31–33^. So far it is unclear whether UPS-mediated protein degradation also plays a role in maintaining PKP2 protein at physiological levels in CMs. While here we show therapeutic benefit in a mutant *Pkp2* mouse model, another recent study utilized gene therapy to correct the arrhythmic phenotype observed in a mouse model of ACM carrying a homozygous knock-in variant in the *Dsg2* gene (*Dsg2*^−/−^)^34^. The authors were able to rescue the arrhythmic but not the fibrotic phenotype in the mutant mice, by AAV9-mediated administration of the truncated isoform of CX43 (*GJA1-20k*), which is responsible for correct localization of CX43 at the IDs.

In this study, we used murine models of PKP2 haploinsufficiency harboring the mouse equivalent of the known pathogenic mutation *PKP2* c.2013delC to mimic the genetic condition of patients. However, there are significant differences between the electrophysiological properties of hearts of human and mice^35^. For example, mouse hearts have weaker Ca^2+^ currents and stronger K^+^ currents^36^ resulting in a shorter ventricular action potential duration and a heart rate about ten times higher than in humans. These differences, including the sparsity of detectable T-waves in mouse hearts^37^, complicate the identification of cardiac defects in our models.

In vitro, we employed EHM as a valuable tool to study cardiac function. Nevertheless, EHM have their own set of limitations when it comes to modeling arrhythmias. Arrhythmias in ACM often coincide with the presence of abnormal collagen deposition and lipid droplets. In our *PKP2* mutant tissues, we did not observe either of these characteristics. This observation aligns with expectations since these changes typically take years to develop in patients with ACM, whereas our tissue samples were cultured for only 1 month. Additionally, it is worth noting that only the CMs in our model were patient-specific, while the cardiac fibroblasts were not. There is an ongoing debate in the scientific literature regarding whether arrhythmias are primarily driven by CMs alone or in combination with other cell types^38–40^. Many studies suggest that factors such as fibrosis, CM death and the infiltration of fibro-fatty tissue create the substrate for triggering arrhythmias^41^. However, there is also evidence indicating that arrhythmias in ACM can manifest before structural abnormalities become apparent^42^. Notably, our 2D iPS-cell-derived CM cultures exhibited sodium conduction irregularities, indicating the presence of a proarrhythmic substrate in our models that may require additional triggers for arrhythmia manifestation (Fig. 2b,c).

So far cardiac gene therapy has been challenging due to a relatively low targeting efficiency owing to the complex architecture of the heart, being composed of multiple cell types. Despite the predominantly non-integrative nature of AAV vectors^43^, multi-year transgene expression after gene transfer has been documented in large animals and humans^44–46^. The fact that we can detect the MYC epitope signal in murine CMs 10 months after the virus administration might be indicating that the promising ‘one and done’ AAV strategy employed in mice could potentially be feasible in human patients as well. In addition, our results indicate that PKP2 restoration in almost 70% of the CMs of the murine ventricles is sufficient to lead to a functional benefit in the mutant animals (Extended Data Fig. 6a,b). While AAV-based gene therapies have been shown to target the liver^29^, our data indicated nonsignificant levels of exogenous PKP2 in the lung, spleen and kidneys of mice; however, moderate expression of cardiac PKP2 in the liver was observed (Extended Data Fig. 6d,e). Should it be desirable, liver targeting can be avoided by the use of a cardiac-specific promoter to drive *PKP2* expression, the use of a cardiotropic capsid and the addition of a liver detargeting sequence. For example, AAV2i8, AAV2i8G9 and AAV-SASTG chimeras, some AAV serotype 9 variants, or vectors obtained through the screening of peptide display libraries or DNA-shuffled libraries^47,48^ all display improved cardio tropism. A cardiotropic capsid (BNP116) obtained as an AAV2/AAV8 chimera^49^ is currently used in a gene therapy clinical trial for heart failure^50^.

ACM is an inherited heart condition characterized by progressive structural changes in the myocardium, which increase the risk of arrhythmias and impair contractile function. In the absence of curative options, current therapeutic interventions for patients suffering from ACM are aimed at controlling disease progression and include antiarrhythmic medications, use of implantable cardioverter defibrillators, catheter ablation and often lifestyle changes^51^. Approaches that interfere with the underlying cause of the disease, in this case PKP2 haploinsufficiency, could potentially be more efficacious in halting or reversing the disease course. However, identification of the patient population that would benefit from *PKP2* gene therapy is currently challenging. Genetic testing could help to identify patients that could suffer from PKP2 haploinsufficiency^52^. However, this type of testing is usually reserved for individuals with a family history of cardiac disease or for those who have already been diagnosed with a cardiac condition and does not necessarily indicate what is happening at the protein level. Readouts in non-cardiac cells that express desmosomal proteins might provide a non-invasive tool to determine cardiac desmosomal protein content indirectly. Researchers have detected a significant reduction of JUP protein in buccal mucosa cells of patients with ACM compared to healthy counterparts^53,54^. Additionally, the PKP2 levels in keratinocytes has been shown to mirror the cardiac level of PKP2^13^. Such a non-invasive, quantitative method to assess cardiac levels of PKP2, combined with a comprehensive evaluation by a healthcare provider, could effectively identify patients that would benefit from *PKP2* gene replacement therapy.

So far, six AAV gene therapy products have already been approved for clinical use for non-cardiac indications, with over 1,400 patients having already been treated with onasemnogene^55^, an intravenously administered AAV9 vector expressing the survival of motor neuron 1 (*SMN1*) protein for spinal muscular atrophy (SMA). After the initial drawback of the AAV1/*SERCA2α* gene therapy for heart failure, which failed in a large phase IIb clinical trial^56^, gene therapy for the heart is also picking up speed again. AskBio takes advantage of a cardiotropic AAV vector with a chimeric AAV2/AAV8 capsid (BNP116)^49^ to overexpress a constitutively active form of Inhibitor-1c (*I-1c*) in patients with heart failure (NCT04179643). Earlier this year, Rocket Pharmaceuticals has received regenerative medicine advanced therapy designation for an AAV9-based gene therapy to express the B isoform of the lysosomal associated membrane protein-2 (*LAMP-2*) to tackle Danon disease after the success of a phase I clinical trial (NCT03882437). On top of that, in May 2023 the same company received an Investigational New Drug (IND) approval for clinical gene therapy for *PKP2* for ACM patients, a highly promising development for the field’s future. Tenaya Therapeutics has received fast track designation for a phase 1b clinical trial for *MYBPC3* gene replacement therapy for hypertrophic cardiomyopathy (NCT05836259), utilizing an optimized AAV9 vector to package the full-length *MYBPC3* gene. In addition, the company received orphan drug designation to its *PKP*2 gene therapy product candidate TN-401 for treatment in 2022 with preclinical results in preparation and a filing for IND in 2023.

Overall, the potential of gene therapy in treating genetic diseases represents a paradigm shift in the way we approach these conditions. Although *PKP2* gene replacement therapy has demonstrated promise in preclinical studies using our knock-in mice, additional studies in suitable models are necessary to establish appropriate dosing regimens and determine potential safety issues. Heterogeneity in targeting of individual CMs could render the heart more susceptible to arrhythmias. A meticulous assessment of the risk of arrhythmias demands rigorous testing in larger animal models that accurately mimic human cardiac physiology in terms of heart rates, size, and function.

To conclude, our data support the notion that *PKP2* gene therapy holds promise for improving the clinical outcomes of patients with ACM with PKP2 haploinsufficiency, and reinforce the promise of gene therapy for tackling heart disease.

## Methods

### Human iPS cell lines

The human *PKP2* c.2013delC and *PKP2* c.1849C>T iPS cell lines were provided by H.-S. V. Chen at University of California San Diego^40^ and J. Wu at Stanford Cardiovascular Institute (supported by National Institutes of Health R24 HL117756), respectively.

### Cell culture

Human iPS cells were grown on Geltrex LDEV-Free, hESC-Qualified, Reduced Growth Factor Basement Membrane Matrix-coated wells (Gibco, A1413302). The cells received fresh Essential 8 Medium (Gibco, A1517001) on a daily basis and were passaged at 80–100% confluency levels. In brief, medium was aspirated and dissociation of the cells was performed with TrypLE Express Enzyme (Gibco, 12605010) for 5 min at 37 °C. After incubation, 4 ml of Essential 8 Medium, supplemented with 2 μM thiazovivin (Sigma-Aldrich, 420220), was added to the dissociated cells and transferred to a 15 ml Falcon tube. Cells were centrifuged for 3 min at 300*g*. Lastly, cells were seeded at a density of 15,000 cells cm^−^^2^ in Essential 8 Medium, supplemented with 2 μM thiazovivin. Medium was refreshed the next day with plain Essential 8.

### CM differentiation

The differentiation protocol started when human iPS cells reached 80–90% of confluency (day 0). Cells were fed with RPMI-1640-Medium-GlutaMAX Supplement-HEPES (Gibco, 72400-021) supplemented with 0.5 mg ml^−1^ human recombinant albumin (Sigma-Aldrich, A9731), 0.2 mg ml^−1^ l-ascorbic acid 2-phosphate (Sigma-Aldrich, A8960) and 4 μM CHIR99021 (Sigma-Aldrich, 361559). After 48 h (day 2), medium was replaced by RPMI-1640-Medium-GlutaMAX Supplement-HEPES supplemented with 0.5 mg ml^−1^ human recombinant albumin (Sigma-Aldrich, A9731), 0.2 mg ml^−1^ l-ascorbic acid 2-phosphate and 5 μM IWP2 (Sigma-Aldrich, 681671). On day 4 and day 6, cells were refreshed with RPMI-1640-Medium-GlutaMAX Supplement-HEPES supplemented with 0.5 mg ml^−1^ human recombinant albumin and 0.2 mg ml^−1^ l-ascorbic acid 2-phosphate. From day 8 onwards, the medium of the cells was refreshed every 3–4 days with RPMI-1640-Medium-GlutaMAX Supplement-HEPES supplemented with B-27 Supplement (50×)-serum free (Gibco, 17504001).

### CM purity

To assess the purity, 1 × 10^6^ iiPS-cell-derived CMs at 15 days of age were utilized. The CMs were subjected to centrifugation at 300*g* for 5 min. The medium was removed, and cells were washed with Dulbecco’s Phosphate-Buffered Saline (dPBS) (Gibco, 14190094). Following another centrifugation at 300*g* for 5 min, the dPBS was aspirated, and cells were fixed by adding 1 ml of ice-cold 70% ethanol while vortexing. After a subsequent centrifugation at 300*g* for 4 min, the fixative was removed. Permeabilization was achieved by resuspending the cells in blocking buffer consisting of PBS (pH 7.2–7.4), supplemented with 5% fetal bovine serum, 1% bovine serum albumin (Sigma-Aldrich, A9647-100G) and 0.5% Triton X-100 (Sigma‐Aldrich, 93443). Following a 10-min incubation at 4 °C, permeabilized cells underwent another centrifugation for 4 min at 300*g*, and the supernatant was aspirated. The cell pellet was resuspended in 100 μl of blocking buffer containing anti-Cardiac Troponin T antibody (Abcam, ab45932; 1:2,000) and incubated at 4 °C for 1 h. Subsequently, 500 μl of blocking solution was added, and cells were centrifuged again for 4 min at 300*g*. After aspirating the supernatant, cells were resuspended once more in 500 μl of blocking buffer, followed by another centrifugation step for 4 min at 300*g*. Cells were then resuspended in 100 μl of blocking buffer containing Alexa 488-anti-rabbit antibody (Thermo Fisher Scientific, A-21206; 1:4,000) and incubated for 30 min at room temperature. Next, 500 μl of blocking buffer was added, and cells were centrifuged for 4 min at 300*g*. The supernatant was discarded, and cells were resuspended again with 500 μl of blocking buffer, followed by another centrifugation for 4 min at 300*g*. Finally, the supernatant was aspirated, and cells were resuspended in 1 ml of dPBS for analysis by fluorescence-activated cell sorting (BD Biosciences, FACS Calibur).

### Automated patch clamp

iPS-cell-derived CM collection proceeded with a PBS wash followed by a Versene wash before dissociation with TrypLE Express (all: Gibco) for 10 min at 37 °C. The cells were resuspended in divalent-free HBSS (Gibco) at 4 °C before measurement. Automated patch clamp experiments were conducted with the SyncroPatch 384 (Nanion Technologies GmbH) device with thin borosilicate glass, single aperture 384-well chips (NPC384T 1× S-type). Application of negative pressure (150–250 mbar) attained whole-cell configuration. *I*_Na_ recordings were performed at 0.5 Hz using a voltage step protocol with a holding potential of −80 mV followed by a hyperpolarizing step to −110 mV for 100 ms and a 300-ms test pulse to −20 mV. Pipette solution contained (in mmol l^−1^): egtazic acid 10, HEPES 10, KCl 10, NaCl 10 and KF 110, pH 7.2 (with KOH). Bath solution contained (in mmol l^−1^): HEPES 10, NaCl 80, N-methyl-d-glucamine (NMDG) 60, glucose 5, KCl 4, CaCl_2_ 2 and MgCl_2_ 1, pH 7.4 at 22–24 °C (with KOH). Currents were recorded with an integrated amplifier controlled by PatchControl 384 software and analyzed offline using DataControl 384 software (both: Nanion Technologies GmbH)^57^.

### EHM generation

EHM was generated according to the protocol published by Tiburcy et al.^58^. In brief, patient-derived iPS-cell-derived CMs (purity >90%) were mixed together with HFFs (HFF-1, ATCC, SCRC-1041) at a ratio of 70:30. The cell mixture was resuspended in an appropriate volume of Collagen type I (Collagen Solutions, FS22024) diluted into RPMI 2× (Thermo Fisher Scientific, 51800-035). A total of 185 μl of the cell–collagen mixture was cast in each well of a 48 EHM multi-well plate (myrPlate-TM5; myriamed GmbH). The cast mixture was incubated for approximately 45 min at 37 °C and subsequently EHM medium freshly supplemented with TGFβ1 (Peprotech, AF-100-21C) was added. During the initial 3 days following the casting process, tissue medium was refreshed daily with EHM medium supplemented with TGFβ1. Subsequently, the tissue medium was replaced daily with EHM medium for the entirety of the experimental duration.

### Contraction analyses

Contraction measurements were performed using video-optic recordings of EHM mediated pole bending in a myrPlate-TM5 culture format at 37 °C (ref. ^59^). Data were recorded from spontaneously contraction EHM for at least 2 min at 50 fps at the indicated time points in a myrImager prototype (myriamed GmbH). Percent pole bending is reported as a surrogate for force of contraction (*F*); contraction and relaxation times are recorded from 20% to 80% peak contraction and 20% to 80% relaxation; contraction and relaxation velocities are reported as maximal and mininmal d*F*/d*t*.

### Quantitative real-time PCR

For iPS-cell-derived CMs, RNA isolation was performed utilizing the RNeasy kit (Qiagen, 74104) as per the manufacturer’s guidelines. Complementary DNA synthesis was conducted using the iScript cDNA Synthesis Kit (Bio-Rad). Quantitative PCR analysis was carried out using the CFX96 Realtime PCR system (Bio-Rad) and iQ SYBR Green (Bio-Rad) in accordance with the manufacturers’ instructions (Supplementary Table 1).

### Mouse line generation

All animal studies carried out in this research adhered to the institutional guidelines and complied with the regulations set forth by the Animal Welfare Committee of the Royal Netherlands Academy of Arts and Sciences. Animal experiments were conducted upon approval by the ‘Animal Welfare Body Utrecht’ (I.v.D.) of the Royal Dutch Academy of Sciences and Arts (K.N.A.W.) and are in compliance with national legislation and institutional guidelines.

Mouse lines were maintained on C57B/6J background. Male and female mice were included in studies performed in pups, whereas studies in adult mice only included males.

### Echocardiographic analysis

Isoflurane-anesthetized mice were subjected to transthoracic M-mode echocardiographic recordings while placed on a heat mat. The recordings were conducted using a Visual Sonic Ultrasound System connected to a 30 MHz transducer. For each mouse, three measurements were taken for various cardiac parameters including LVEDV, LVESV, end-diastolic interventricular septal wall thickness, end-systolic interventricular septal wall thickness, IVRT and the early (E) and late (A) ventricular filling velocities. The E and A values were utilized to calculate the E/A ratio. Cardiac function, specifically EF (%), was automatically determined by the software using the averaged values of the aforementioned parameters.

### Histology and immunohistochemistry

Cardiac tissue from mice or EHM was collected and briefly washed in ice-cold PBS. After rinsing and weighing, they were fixed in 4% paraformaldehyde for 48 or 24 h, respectively, at room temperature. The fixed tissues were then embedded in paraffin and sliced at 4 μm. The sections were dewaxed and rehydrated. For immunohistochemistry, tissue sections were boiled for 20 min in either ethylenediaminetetraacetic acid (EDTA) buffer or sodium citrate buffer. They were then blocked for 45 min at room temperature using a solution containing 0.1% bovine serum albumin and 0.4% TWEEN20 dissolved in dPBS. The tissue was then incubated with primary antibodies overnight at 4 °C, followed by incubation with secondary antibodies for 1 h at room temperature. DAPI diluted in dPBS (1:1,000) was used to stain the sections, and they were subsequently mounted with Mowiol and imaged using a Leica TCS SPE confocal microscope. Leica Application Suite (LAS X, version 3.30 or newer) was used for image acquisition, whereas image processing was performed with Fiji. Supplementary Table 2 presents information about all antibodies used for immunofluorescence assays.

### Western blot

iPS-cell-derived CMs were dissociated using TrypLE Select Enzyme (10×) and collected in a 1.5-ml Eppendorf tube. The cells were then centrifuged at 300*g* for 5 min and resuspended in 1 ml of dPBS, followed by another centrifugation round with the same conditions. The cells were then lysed in RIPA buffer containing cOmplete EDTA-free Protease Inhibitor Cocktail (one tablet per 10 ml of RIPA buffer) and PhosSTOP (one tablet per 10 ml of RIPA buffer). For immunoblotting, 10–15 μg of protein extract was used. Horseradish peroxidase-coupled secondary antibodies were used in combination with the Clarity Western ECL Substrate kit for visualization. Immunoblots were imaged using an Amersham Imager 680RGB device and quantified with ImageQuant TL software v7.1 (GE Healthcare).

Immunoblotting was also performed on protein lysates from snap-frozen mouse tissue explanted from the ventricles. The tissue was lysed in approximately 150 μl of RIPA buffer as described above. Immunoblotting was performed using 20–50 μg of protein. In Supplementary Table 3 there are all details regarding antibodies used.

### AAV delivery

AAV vectors (serotype 6 or 9) encoding human *PKP2*, murine *Pkp2* or empty vector were generated in collaboration with the Giacca and Zentilin labs (Trieste, Italy).

We used 5 × 10^3^ v.g. per cell to infect hiPS-cell-derived CMs with AAV6. AAV9-ctr and AAV9–PKP2 were used for in vivo studies. A total of 3 × 10^11^ v.g. per animal were injected intraperitoneally into 5-day-old pups using an insulin syringe with a 30-gauge needle. Adult mice were injected intravenously through the tail vein with 5 × 10^12^ v.g. per animal (one single injection) using a syringe with a 26-gauge needle.

### Statistical analysis

Data are presented as mean ± standard error of the mean. Statistical differences between two groups were tested by two-sided unpaired or paired Student’s *t*-tests. In case of three and more groups, one-way or two-way unrepeated or repeated-measures analysis of variance (ANOVA) with appropriate post-hoc testing was performed. The performed tests are specified in the respective figure legends. Statistical testing was performed with GraphPad Prism 9.5.1.

### Reporting summary

Further information on research design is available in the Nature Portfolio Reporting Summary linked to this article.

## Supplementary information

## Source data

## Data Availability

All data supporting the findings in this study are available within the paper and associated files. Source data are provided with this paper.

## References

[1] Pilichou, K. et al. Arrhythmogenic cardiomyopathy. *Orphanet J. Rare Dis.* **11**, 33 (2016). 27038780 10.1186/s13023-016-0407-1 PMC4818879

[2] Sen-Chowdhry, S. et al. Mutational heterogeneity, modifier genes, and environmental influences contribute to phenotypic diversity of arrhythmogenic cardiomyopathy. *Circ. Cardiovasc. Genet.* **3**, 323–330 (2010). 20570917 10.1161/CIRCGENETICS.109.935262

[3] Basso, C., Corrado, D., Bauce, B. & Thiene, G. Arrhythmogenic right ventricular cardiomyopathy. *Circ. Arrhythm. Electrophysiol.* **5**, 1233–1246 (2012). 23022706 10.1161/CIRCEP.111.962035

[4] Giuliano, K. et al. Heart transplantation outcomes in arrhythmogenic right ventricular cardiomyopathy: a contemporary national analysis. *ESC Heart Fail.* **9**, 988–997 (2022). 35132806 10.1002/ehf2.13687 PMC8934952

[5] Marcus, F. I. et al. Diagnosis of arrhythmogenic right ventricular cardiomyopathy/dysplasia: proposed modification of the Task Force Criteria. *Eur. Heart J.* **31**, 806–814 (2010). 20172912 10.1093/eurheartj/ehq025 PMC2848326

[6] Vermij, S. H., Abriel, H. & van Veen, T. A. Refining the molecular organization of the cardiac intercalated disc. *Cardiovasc. Res.* **113**, 259–275 (2017). 28069669 10.1093/cvr/cvw259

[7] van Hengel, J. et al. Mutations in the area composita protein αT-catenin are associated with arrhythmogenic right ventricular cardiomyopathy. *Eur. Heart J.* **34**, 201–210 (2013). 23136403 10.1093/eurheartj/ehs373

[8] Agullo-Pascual, E., Cerrone, M. & Delmar, M. Arrhythmogenic cardiomyopathy and Brugada syndrome: diseases of the connexome. *FEBS Lett.* **588**, 1322–1330 (2014). 24548564 10.1016/j.febslet.2014.02.008 PMC3989410

[9] Sato, P. Y. et al. Interactions between ankyrin-G, Plakophilin-2, and Connexin43 at the cardiac intercalated disc. *Circ. Res.* **109**, 193–201 (2011). 21617128 10.1161/CIRCRESAHA.111.247023 PMC3139453

[10] Jacob, K. A. et al. Geographical distribution of plakophilin-2 mutation prevalence in patients with arrhythmogenic cardiomyopathy. *Neth. Heart J.* **20**, 234–239 (2012). 22527912 10.1007/s12471-012-0274-x PMC3346879

[11] Basharat, S. A., Hsiung, I., Garg, J. & Alsaid, A. Arrhythmogenic cardiomyopathy: evolving diagnostic criteria and insight from cardiac magnetic resonance imaging. *Heart Fail. Clin.* **19**, 429–444 (2023). 37714585 10.1016/j.hfc.2023.03.006

[12] Hylind, R. J. et al. Population prevalence of premature truncating variants in plakophilin-2 and association with arrhythmogenic right ventricular cardiomyopathy: a UK Biobank analysis. *Circ. Genom. Precis. Med.* **15**, e003507 (2022). 35536239 10.1161/CIRCGEN.121.003507 PMC9400410

[13] Rasmussen, T. B. et al. Truncating plakophilin-2 mutations in arrhythmogenic cardiomyopathy are associated with protein haploinsufficiency in both myocardium and epidermis. *Circ. Cardiovasc. Genet.* **7**, 230–240 (2014). 24704780 10.1161/CIRCGENETICS.113.000338

[14] Zhang, K. et al. Plakophilin-2 truncating variants impair cardiac contractility by disrupting sarcomere stability and organization. *Sci. Adv.* **7**, eabh3995 (2021). 34652945 10.1126/sciadv.abh3995 PMC8519574

[15] Cerrone, M. et al. Plakophilin-2 is required for transcription of genes that control calcium cycling and cardiac rhythm. *Nat. Commun.* **8**, 106 (2017). 28740174 10.1038/s41467-017-00127-0 PMC5524637

[16] Goossens, S. et al. A unique and specific interaction between αT-catenin and plakophilin-2 in the area composita, the mixed-type junctional structure of cardiac intercalated discs. *J. Cell Sci.* **120**, 2126–2136 (2007). 17535849 10.1242/jcs.004713

[17] Pérez-Hernández, M. et al. Transcriptomic coupling of PKP2 with inflammatory and immune pathways endogenous to adult cardiac myocytes. *Front. Physiol.* **11**, 623190 (2020). 33536940 10.3389/fphys.2020.623190 PMC7849609

[18] Dubash, A. D. et al. Plakophilin-2 loss promotes TGF-β1/p38 MAPK-dependent fibrotic gene expression in cardiomyocytes. *J. Cell Biol.* **212**, 425–438 (2016). 26858265 10.1083/jcb.201507018 PMC4754716

[19] Liang, Y. et al. Desmosomal COP9 regulates proteome degradation in arrhythmogenic right ventricular dysplasia/cardiomyopathy. *J. Clin. Invest.* **131**, e137689 (2021). 33857019 10.1172/JCI137689 PMC8159691

[20] Tsui, H. et al. Desmosomal protein degradation as an underlying cause of arrhythmogenic cardiomyopathy. *Sci. Transl. Med.* **15**, eadd4248 (2023). 36947592 10.1126/scitranslmed.add4248

[21] Pérez-Hernández, M. et al. Loss of nuclear envelope integrity and increased oxidant production cause DNA damage in adult hearts deficient in PKP2: a molecular substrate of ARVC. *Circulation* **146**, 851–867 (2022). 35959657 10.1161/CIRCULATIONAHA.122.060454 PMC9474627

[22] van Opbergen, C. J. M. et al. Plakophilin-2 haploinsufficiency causes calcium handling deficits and modulates the cardiac response towards stress. *Int. J. Mol. Sci.* **20**, 4076 (2019). 31438494 10.3390/ijms20174076 PMC6747156

[23] Peters, S. Editorial: cardiomyopathies: current treatment and future options. *J. Clin. Med.* **9**, 3531 (2020). 33142802 10.3390/jcm9113531 PMC7693478

[24] Bosman, L. P. et al. The Netherlands Arrhythmogenic Cardiomyopathy Registry: design and status update. *Neth. Heart J.* **27**, 480–486 (2019). 30997596 10.1007/s12471-019-1270-1 PMC6773794

[25] Groeneweg, J. A. et al. Clinical presentation, long-term follow-up, and outcomes of 1001 arrhythmogenic right ventricular dysplasia/cardiomyopathy patients and family members. *Circ. Cardiovasc. Genet.* **8**, 437–446 (2015). 25820315 10.1161/CIRCGENETICS.114.001003

[26] Cerrone, M. et al. Sodium current deficit and arrhythmogenesis in a murine model of plakophilin-2 haploinsufficiency. *Cardiovasc. Res.* **95**, 460–468 (2012). 22764151 10.1093/cvr/cvs218 PMC3422082

[27] Shaw, R. M. Reduced sodium channels in human ARVC. *Heart Rhythm* **10**, 420–421 (2013). 23266405 10.1016/j.hrthm.2012.12.019 PMC4279716

[28] Tiburcy, M., Meyer, T., Satin, P. L. & Zimmermann, W. H. Defined engineered human myocardium for disease modeling, drug screening, and heart repair. *Methods Mol. Biol.* **2485**, 213–225 (2022). 35618908 10.1007/978-1-0716-2261-2_14

[29] Zincarelli, C., Soltys, S., Rengo, G. & Rabinowitz, J. E. Analysis of AAV serotypes 1–9 mediated gene expression and tropism in mice after systemic injection. *Mol. Ther.* **16**, 1073–1080 (2008). 18414476 10.1038/mt.2008.76

[30] Inoue, H. et al. Modeling reduced contractility and impaired desmosome assembly due to plakophilin-2 deficiency using isogenic iPS cell-derived cardiomyocytes. *Stem Cell Rep.* **17**, 337–351 (2022). 10.1016/j.stemcr.2021.12.016 PMC8828557 35063130

[31] Tardiff, J. C. et al. Targets for therapy in sarcomeric cardiomyopathies. *Cardiovasc. Res.* **105**, 457–470 (2015). 25634554 10.1093/cvr/cvv023 PMC4402369

[32] Prondzynski, M. et al. Evaluation of MYBPC3 *trans*-splicing and gene replacement as therapeutic options in human iPSC-derived cardiomyocytes. *Mol. Ther. Nucleic Acids* **7**, 475–486 (2017). 28624223 10.1016/j.omtn.2017.05.008 PMC5458066

[33] Mearini, G. et al. Mybpc3 gene therapy for neonatal cardiomyopathy enables long-term disease prevention in mice. *Nat. Commun.* **5**, 5515 (2014). 25463264 10.1038/ncomms6515

[34] Palatinus, J. A. et al. GJA1-20k rescues Cx43 localization and arrhythmias in arrhythmogenic cardiomyopathy. *Circ. Res.* **132**, 744–746 (2023). 36927183 10.1161/CIRCRESAHA.122.322294 PMC10314823

[35] Clauss, S. et al. Animal models of arrhythmia: classic electrophysiology to genetically modified large animals. *Nat. Rev. Cardiol.* **16**, 457–475 (2019). 30894679 10.1038/s41569-019-0179-0

[36] Gussak, I., Chaitman, B. R., Kopecky, S. L. & Nerbonne, J. M. Rapid ventricular repolarization in rodents: electrocardiographic manifestations, molecular mechanisms, and clinical insights. *J. Electrocardiol.* **33**, 159–170 (2000). 10819409 10.1016/s0022-0736(00)80072-2

[37] Liu, G. et al. In vivo temporal and spatial distribution of depolarization and repolarization and the illusive murine T wave. *J. Physiol.* **555**, 267–279 (2004). 14634200 10.1113/jphysiol.2003.054064 PMC1664824

[38] Sommariva, E. et al. Cardiac mesenchymal stromal cells are a source of adipocytes in arrhythmogenic cardiomyopathy. *Eur. Heart J.* **37**, 1835–1846 (2016). 26590176 10.1093/eurheartj/ehv579 PMC4912024

[39] Caspi, O. et al. Modeling of arrhythmogenic right ventricular cardiomyopathy with human induced pluripotent stem cells. *Circ. Cardiovasc. Genet.* **6**, 557–568 (2013). 24200905 10.1161/CIRCGENETICS.113.000188

[40] Kim, C. et al. Studying arrhythmogenic right ventricular dysplasia with patient-specific iPSCs. *Nature* **494**, 105–110 (2013). 23354045 10.1038/nature11799 PMC3753229

[41] Asimaki, A., Kleber, A. G. & Saffitz, J. E. Pathogenesis of arrhythmogenic cardiomyopathy. *Can. J. Cardiol.* **31**, 1313–1324 (2015). 26199027 10.1016/j.cjca.2015.04.012 PMC4619183

[42] Gomes, J. et al. Electrophysiological abnormalities precede overt structural changes in arrhythmogenic right ventricular cardiomyopathy due to mutations in desmoplakin-A combined murine and human study. *Eur. Heart J.* **33**, 1942–1953 (2012). 22240500 10.1093/eurheartj/ehr472 PMC3409421

[43] Li, H. et al. Assessing the potential for AAV vector genotoxicity in a murine model. *Blood* **117**, 3311–3319 (2011). 21106988 10.1182/blood-2010-08-302729 PMC3069673

[44] Nathwani, A. C. et al. Long-term safety and efficacy following systemic administration of a self-complementary AAV vector encoding human FIX pseudotyped with serotype 5 and 8 capsid proteins. *Mol. Ther.* **19**, 876–885 (2011). 21245849 10.1038/mt.2010.274 PMC3098629

[45] Nathwani, A. C. et al. Long-term safety and efficacy of factor IX gene therapy in hemophilia B. *N. Engl. J. Med.* **371**, 1994–2004 (2014). 25409372 10.1056/NEJMoa1407309 PMC4278802

[46] Niemeyer, G. P. et al. Long-term correction of inhibitor-prone hemophilia B dogs treated with liver-directed AAV2-mediated factor IX gene therapy. *Blood* **113**, 797–806 (2009). 18957684 10.1182/blood-2008-10-181479 PMC2630266

[47] Zacchigna, S., Zentilin, L. & Giacca, M. Adeno-associated virus vectors as therapeutic and investigational tools in the cardiovascular system. *Circ. Res.* **114**, 1827–1846 (2014). 24855205 10.1161/CIRCRESAHA.114.302331

[48] Grimm, D. & Büning, H. Small but increasingly mighty: latest advances in AAV vector research, design, and evolution. *Hum. Gene Ther.* **28**, 1075–1086 (2017). 28835125 10.1089/hum.2017.172

[49] Ishikawa, K. et al. Cardiac I-1c overexpression with reengineered AAV improves cardiac function in swine ischemic heart failure. *Mol. Ther.* **22**, 2038–2045 (2014). 25023328 10.1038/mt.2014.127 PMC4429688

[50] NAN-101 in patients with class III heart failure (NAN-CS101). *National Library of Medicine* https://clinicaltrials.gov/ct2/show/NCT04179643. (2019).

[51] Migliore, F. et al. Arrhythmogenic cardiomyopathy—current treatment and future options. *J. Clin. Med.* **10**, 2750 (2021). 34206637 10.3390/jcm10132750 PMC8268983

[52] de Brouwer, R. et al. Value of genetic testing in the diagnosis and risk stratification of arrhythmogenic right ventricular cardiomyopathy. *Heart Rhythm* **19**, 1659–1665 (2022). 35688345 10.1016/j.hrthm.2022.05.038

[53] Asimaki, A. et al. Characterizing the molecular pathology of arrhythmogenic cardiomyopathy in patient buccal mucosa cells. *Circ. Arrhythm. Electrophysiol.* **9**, e003688 (2016). 26850880 10.1161/CIRCEP.115.003688 PMC4785796

[54] Driessen, H. E. et al. Buccal mucosa cells as a potential diagnostic tool to study onset and progression of arrhythmogenic cardiomyopathy. *Int. J. Mol. Sci.* **23**, 57 (2021). 35008484 10.3390/ijms23010057 PMC8744793

[55] Aslesh, T. & Yokota, T. Restoring SMN expression: an overview of the therapeutic developments for the treatment of spinal muscular atrophy. *Cells* **11**, 417 (2022). 35159227 10.3390/cells11030417 PMC8834523

[56] Greenberg, B. et al. Calcium upregulation by percutaneous administration of gene therapy in patients with cardiac disease (CUPID 2): a randomised, multinational, double-blind, placebo-controlled, phase 2b trial. *Lancet* **387**, 1178–1186 (2016). 26803443 10.1016/S0140-6736(16)00082-9

[57] Seibertz, F. et al. A modern automated patch-clamp approach for high throughput electrophysiology recordings in native cardiomyocytes. *Commun. Biol.* **5**, 969 (2022). 36109584 10.1038/s42003-022-03871-2 PMC9477872

[58] Tiburcy, M. et al. Defined engineered human myocardium with advanced maturation for applications in heart failure modeling and repair. *Circulation* **135**, 1832–1847 (2017). 28167635 10.1161/CIRCULATIONAHA.116.024145 PMC5501412

[59] Tiburcy, M., Meyer, T., Liaw, N. Y. & Zimmermann, W. H. Generation of engineered human myocardium in a multi-well format. *STAR Protoc.* **1**, 100032 (2020). 33111083 10.1016/j.xpro.2020.100032 PMC7580201

