# Supplementary material for: Therapeutic efficacy of AAV-mediated restoration of PKP2 in arrhythmogenic cardiomyopathy
Source: Nat Cardiovasc Res. 2023 Dec 7;2(12):1262–76. doi: 10.1038/s44161-023-00378-9 (PMC11041734; doi:10.1038/s44161-023-00378-9)
Supplement: Supplementary file 1 — Tables 1–3 [file 44161_2023_378_MOESM1_ESM.pdf]

nature cardiovascular research

Article https://doi.org/10.1038/s44161-023-00378-9

Therapeutic efficacy of AAV-mediated restoration of PKP2 in arrhythmogenic cardiomyopathy

In the format provided by the authors and unedited

Table S1: Quantitative PCR primers

Gene (Species)	Forward sequence (5'->3')	Reverse sequence (5'->3')
PKP2 (Human)	TGCTAAAGGCTGGCACAA	TAATCGCTGTGCGTGTAGTG
JUP (Human)	ACCAGGAGAGCAAGCTGAT	CTCCACAATGGCAGGCTTATT
DSP (Human)	GCACCAGCAGGATGTACTATT	TCAATTCAGGCTGCACGAT
DSC (Human)	TGGTAGAGTTAACCTGAAAGAGTG	TGGTTCTCAGTGTTGGAAAGT
DSG (Human)	GGAACACAGCAGCTACACTT	ACCATCCCTTCAAGCACTTTAT
ACTN2 (Human)	AGTCCTGACGAGAGATGCG	GGGTCATAATGCGGGCAAAT
VIM (Human)	CGTCAGCAATATGAAAGTGTGTGG	GTCTCCGGTACTCAFTFFACTC
DCN (Human)	TGCAGCTAGCCTGAAAGGAC	TTGGCCAGAGAGCCATTGTC
NPPB (Human)	CTCCAGAGACATGGATCCCC	GTTGCGCTGCTCCTGTAAC
GUS (Human)	CCACCTAGAATCTGCTGGCTAC	GTGCCCGTAGTCGTGATACCAA

Table S2: Antibodies used for Immunofluorescence

Antibody	Catalogue number	Company	Secondary antibody	Antigen Retrieval	Dilution
Plakophilin 2 (PKP2)	ab189323	Abcam	Goat	EDTA	1:100
MYC epitope tag (MYC)	2276S	Cell Signaling Technology	Mouse	EDTA	1:100
A Actinin (ACTN2)	HPA008315	Sigma Aldrich	Rabbit	EDTA	1:100
Vimentin (VIM)	sc-373717	Santa Cruz	Mouse	EDTA	1:250
Desmin (DES)	AF3844	R&D Systems	Goat	EDTA	1:50
Desmoplakin (DSP)	ab71690	Abcam	Rabbit	Citrate	1:100

β catenin (β-CAT)		Cell Signaling Technology	Rabbit	EDTA	1:100
tdTomato	Ab8181	SciGen	Goat	EDTA	1:100

Table S3: Antibodies used for western blot

**Antibody**	**Catalogue number**	**Company**	**Secondary antibody**	**Dilution**
Plakophilin 2 (PKP2)	ab189323	Abcam	Goat	1:800
Plakophilin 2 (PKP2)	610788	BD Transduction Laboratories	Mouse	1:1000
Plakoglobin (JUP)	sc398183	Santa Cruz	Mouse	1:1000
Desmoplakin (DSP)	ab71690	Abcam	Rabbit	1:1000
Desmoglein (DSG)	61002	Progen	Mouse	1:100
Desmocollin (DSC)	32-6200	Thermo Fisher Scientific	Mouse	1:250
MYC epitope tag (MYC)	2276S	Cell Signaling Technology	Mouse	1:1000
N-cadherin (NCAD)	13116S	Cell Signaling Technology	Rabbit	1:1000
α-catenin (α-cat)	C2081	Sigma Aldrich	Rabbit	1:1000
Vinculin (VIN)	sc25336	Santa Cruz	Mouse	1:1000
α-tubulin (α-tub)	T5168	Sigma Aldrich	Mouse	1:1000
Glyceraldehyde 3-phosphate dehydrogenase (GAPDH)	MAB374	Millipore	Mouse	1:5000
