# Supplementary material for: Therapeutic efficacy of AAV-mediated restoration of PKP2 in arrhythmogenic cardiomyopathy
Source: Nat Cardiovasc Res. 2023 Dec 7;2(12):1262–76. doi: 10.1038/s44161-023-00378-9 (PMC11041734; doi:10.1038/s44161-023-00378-9)
Supplement: Supplementary file 2 — Reporting Summary [file 44161_2023_378_MOESM2_ESM.pdf]

nature portfolio

Corresponding author(s): E van Rooij

Last updated by author(s): Oct 27, 2023

Reporting Summary

Nature Portfolio wishes to improve the reproducibility of the work that we publish. This form provides structure for consistency and transparency in reporting. For further information on Nature Portfolio policies, see our Editorial Policies and the Editorial Policy Checklist.

Statistics

For all statistical analyses, confirm that the following items are present in the figure legend, table legend, main text, or Methods section.

n/a	Confirmed	
☐	☒	The exact sample size (*n*) for each experimental group/condition, given as a discrete number and unit of measurement
☐	☒	A statement on whether measurements were taken from distinct samples or whether the same sample was measured repeatedly
☐	☒	The statistical test(s) used AND whether they are one- or two-sided<br>*Only common tests should be described solely by name; describe more complex techniques in the Methods section.*
☒	☐	A description of all covariates tested
☐	☒	A description of any assumptions or corrections, such as tests of normality and adjustment for multiple comparisons
☒	☐	A full description of the statistical parameters including central tendency (e.g. means) or other basic estimates (e.g. regression coefficient) AND variation (e.g. standard deviation) or associated estimates of uncertainty (e.g. confidence intervals)
☐	☒	For null hypothesis testing, the test statistic (e.g. *F*, *t*, *r*) with confidence intervals, effect sizes, degrees of freedom and *P* value noted<br>*Give P values as exact values whenever suitable.*
☒	☐	For Bayesian analysis, information on the choice of priors and Markov chain Monte Carlo settings
☒	☐	For hierarchical and complex designs, identification of the appropriate level for tests and full reporting of outcomes
☒	☐	Estimates of effect sizes (e.g. Cohen's *d*, Pearson's *r*), indicating how they were calculated

Our web collection on statistics for biologists contains articles on many of the points above.

Software and code

Policy information about availability of computer code

Data collection	Leica Application Suite (LAS X, version 3.30 or newer) was used for image acquisition of confocal microscopy data. Chemiluminescence on western blots was recorded using the ImageQuant software v7.1 of the Image Quant LAS 4000 Imaging System (GE Healthcare). Contractile function of EHM has been recorded by the custom-made MyrImager prototype software (MyriaMed GmbH)
Data analysis	Fiji (multiple versions between 2019 and 2023) was used for basic image analysis and adjustment of contrast and brightness. Chemiluminescence signal intensity was quantified using ImageQuant TL software v7.1 (GE Healthcare). Patch Control 384 software was used for the automated single cell patch clamp analysis (Nanion Technologies GmbH). The MyrImager prototype software was used for contraction analyses of the EHM. GraphPad Prism (version 9.5.1) was used for statistical analysis. Vevo Lab version 5.7.1 was used for echocardiography analysis.

For manuscripts utilizing custom algorithms or software that are central to the research but not yet described in published literature, software must be made available to editors and reviewers. We strongly encourage code deposition in a community repository (e.g. GitHub). See the Nature Portfolio guidelines for submitting code & software for further information.

Data

Policy information about availability of data

All manuscripts must include a data availability statement. This statement should provide the following information, where applicable:

- Accession codes, unique identifiers, or web links for publicly available datasets
- A description of any restrictions on data availability
- For clinical datasets or third party data, please ensure that the statement adheres to our policy

All data supporting the findings in this study are available within the paper and associated files. Source data are provided with this manuscript.

Research involving human participants, their data, or biological material

Policy information about studies with human participants or human data. See also policy information about sex, gender (identity/presentation), and sexual orientation and race, ethnicity and racism.

Reporting on sex and gender	n/a
Reporting on race, ethnicity, or other socially relevant groupings	n/a
Population characteristics	n/a
Recruitment	n/a
Ethics oversight	n/a

Note that full information on the approval of the study protocol must also be provided in the manuscript.

Field-specific reporting

Please select the one below that is the best fit for your research. If you are not sure, read the appropriate sections before making your selection.

☒ Life sciences ☐ Behavioural & social sciences ☐ Ecological, evolutionary & environmental sciences

For a reference copy of the document with all sections, see nature.com/documents/nr-reporting-summary-flat.pdf

Life sciences study design

All studies must disclose on these points even when the disclosure is negative.

Sample size	Sample size was predetermined for in vivo studies, using power analysis with type I error (alpha) = 0.05, type II error (beta) = 0.2 and effect size of 10%. For the in vitro studies, no specific sample size calculation was undertaken before experiments. We chose sample size according to previous experience in the field of molecular cardiology, which suggests that robust results have to be reproducible in at least three distinct biological replicates (cardiomyocyte differentiations).
Data exclusions	No data was excluded from the analysis.
Replication	All experiments were performed with a minimal of 3 replicates. Experiments in iPS-CM were performed in multiple (2 to 3) independent differentiations of the same iPS-cell line. The same applies for the studies using EHM tissues, where experiments were performed on tissues coming from 2-3 distinct differentiations. All independent experiments showed similar results, which confirmed reproducibility.
Randomization	Mice were allocated to groups based on their genotype. Where possible, littermate controls were used. Randomization was not relevant for in vitro experiments, however, all cells or samples were treated and analyzed in the same manner across conditions.
Blinding	Investigators were blinded to group allocation during data analysis (echo analysis, gene expression).

Reporting for specific materials, systems and methods

We require information from authors about some types of materials, experimental systems and methods used in many studies. Here, indicate whether each material, system or method listed is relevant to your study. If you are not sure if a list item applies to your research, read the appropriate section before selecting a response.

Materials & experimental systems

n/a	Involved in the study
☐	☒ Antibodies
☐	☒ Eukaryotic cell lines
☒	☐ Palaeontology and archaeology
☐	☒ Animals and other organisms
☒	☐ Clinical data
☒	☐ Dual use research of concern
☒	☐ Plants

Methods

n/a	Involved in the study
☒	☐ ChIP-seq
☒	☐ Flow cytometry
☒	☐ MRI-based neuroimaging

Antibodies

Antibodies used

A complete list with all details about the antibodies used in this study is provided in the supplemental tables 2 and 3. For all antibodies we have provided the supplier name, catalog number and working concentration.

Validation

Antibodies were used as directed by the suppliers. Below some statements from the supplier websites:

Plakophilin 2 (PKP2)-Abcam, ab189323- 1:100 (IF), 1:1000 (WB)-Supplier: Species reactivity include human, rat and mouse. Suitable for WB and IHP.

Anti-c-myc epitope tag [9B11]-Cell Signaling, #2276- 1:100 (IF), 1:1000 (WB)-Supplier: Myc-Tag (9B11) Mouse mAb detects exogenously expressed Myc-tagged proteins in cells expressed under a CMV promoter. Expression under other promoters has not been evaluated. Reactivity with all species.

ACTN2- Sigma Aldrich, HPA008315- 1:100 (IF)-Supplier: All Prestige Antibodies Powered by Atlas Antibodies are developed and validated by the Human Protein Atlas (HPA) project and as a result, are supported by the most extensive characterization in the industry. Species reactivity: human. Applications: Immunohistochemistry.

PKP2- BD Transduction laboratories, 610788- 1:1000 (WB)- Supplier: reactivity-QC testing:human, applications: western blot routinely tested

γ-catenin (D-12)- Santa Cruz, sc398183 - 1:1000 (WB) - Supplier: γ-catenin (D-12) is recommended for detection of γ-catenin of mouse, rat and human origin by Western Blotting (starting dilution 1:100, dilution range1:100-1:1000), immunoprecipitation [1-2 µg per 100-500 µg of total protein (1 ml of cell lysate)], immunofluorescence (starting dilution 1:50, dilutionrange 1:50-1:500) and solid phase ELISA (starting dilution 1:30, dilutionrange 1:30-1:3000).

Anti-desmoplakin I+II - Abcam, ab71690 - 1:1000 (WB) - Supplier: suitable for WB, ICC, IF. Reacts with human.

Desmocollin 2/3 Monoclonal Antibody (7G6) - Invitrogen, # 32-6200, 1:250 (WB), Supplier: This Antibody was verified by Relative expression to ensure that the antibody binds to the antigen stated. Species reactivity include human, mouse. Applications inlcude WB.

anti-Desmoglein 1/2 mouse monoclonal, DG 3.10, lyophilized, purified - Progen, 61002 - 1:100 (WB) - Supplier: Reactivity with bovine, human , rat. Tested applications include WB.

N-Cadherin (D4R1H) XP® Rabbit mAb - Cell Signaling, #13116, 1:1000 (WB) - Supplier: Applications include: WB, IP, IHC-Bond, IHC-P, IF-IC and species reactivity: mouse and human.

Anti-a-Catenin - Sigma Aldrich, C2081 - 1:1000 (WB) - Suppler: Anti-a-Catenin may be used for the immuno-localization of a-catenin by various immunohistochemical methods using frozen tissue sections and cultured cells. It may be used to detect a-catenin by other assays includingdot blot immunoassay and immunoblotting.

α-tubulin (α-tub) - Sigma Aldrich, T5168 - 1:1000 (WB) - Supplier: applications include western blot. Species reactivity include mouse and human.

Vinculin - Santa Cruz, sc-25336 - 1:1000 (WB) - Supplier: Species reactivity include human and mouse. Suitable for WB

β-catenin - Cell Signaling Technology, 8480S - 1:1000 (WB), 1:100 (IF), Species Reactivity: Human, Mouse, Rat (https://www.cellsignal.com/products/primary-antibodies/b-catenin-d10a8-xp-rabbit-mab/8480)

Desmin - https://www.rndsystems.com/products/human-mouse-desmin-antibody_af3844?gad_source=1&gclid=CjwKCAjwnOipBhBQEiwACyGLupYSlEhtHF_5Pe4QHMlPjarG2Ju8Lpeg0t1L_F_iSAHHmBmqFzq3BxoCQVQQAvD_BwE&gclsrc=aw.ds

Vimentin - https://datasheets.scbt.com/sc-373717.pdf

tdTomato - https://www.labome.com/product/SICGEN/AB8181-200.html

Horseradish peroxidase-coupled secondary antibodies - https://www.jacksonimmuno.com/catalog/products/315-035-003 and https://www.jacksonimmuno.com/catalog/products/211-035-109

Anti-Cardiac Troponin T antibody - https://www.abcam.com/en-is/products/primary-antibodies/anti-cardiac-troponin-t-antibody-ab45932

Alexa 488-anti-rabbit antibody - https://www.thermofisher.com/antibody/product/Donkey-anti-Rabbit-IgG-H-L-Highly-Cross-Adsorbed-Secondary-Antibody-Polyclonal/A-21206

Eukaryotic cell lines

Policy information about cell lines and Sex and Gender in Research

Cell line source(s)	The human PKP2 c.2013delC and PKP2 c.1849C>T iPSC lines were provided by H.-S. V. Chen at University of California San Diego45 and J. Wu at Stanford Cardiovascular Institute (supported by National Institutes of Health R24 HL117756), respectively.
Authentication	Authentication of the cells was not performed
Mycoplasma contamination	human iPSC were routinely monitored for mycoplasma contamination and tested negative.
Commonly misidentified lines (See ICLAC register)	No commonly misidentified lines were used.

Animals and other research organisms

Policy information about studies involving animals; ARRIVE guidelines recommended for reporting animal research, and Sex and Gender in Research

Laboratory animals	Mouse lines were maintained on C57B/6J background. PKP2 mutant mice as well.<br>wildtype C57B/6J (Stock#: 000664) mice were obtained from Jackson Laboratories.<br>Mice were housed under normal conditions with food and water ad libitum under normal day and night cycles in monitored environmental conditions. After the virus injection, the animals weree housed in DMI conditions for 2 weeks and until they reach the age of 1 year.
Wild animals	No wild animals were used in this study.
Reporting on sex	Animal studies involving pups included males and females.<br>Animal studies involving adult animals (>8w) included males only.
Field-collected samples	No field-collected data are presented in this study.
Ethics oversight	Animal studies were approved by the animal welfare agency "Animal Welfare Body Utrecht" (IvD) of the Royal Dutch Academy of Sciences and Arts (KNAW) and in compliance with national legislation and institutional guidelines.

Note that full information on the approval of the study protocol must also be provided in the manuscript.
