# Supplementary material for: Therapeutic efficacy of AAV-mediated restoration of PKP2 in arrhythmogenic cardiomyopathy
Source: Nat Cardiovasc Res. 2023 Dec 7;2(12):1262–76. doi: 10.1038/s44161-023-00378-9 (PMC11041734; doi:10.1038/s44161-023-00378-9)
Supplement: Supplementary file 4 — Unprocessed western blot. [file 44161_2023_378_MOESM4_ESM.pdf]

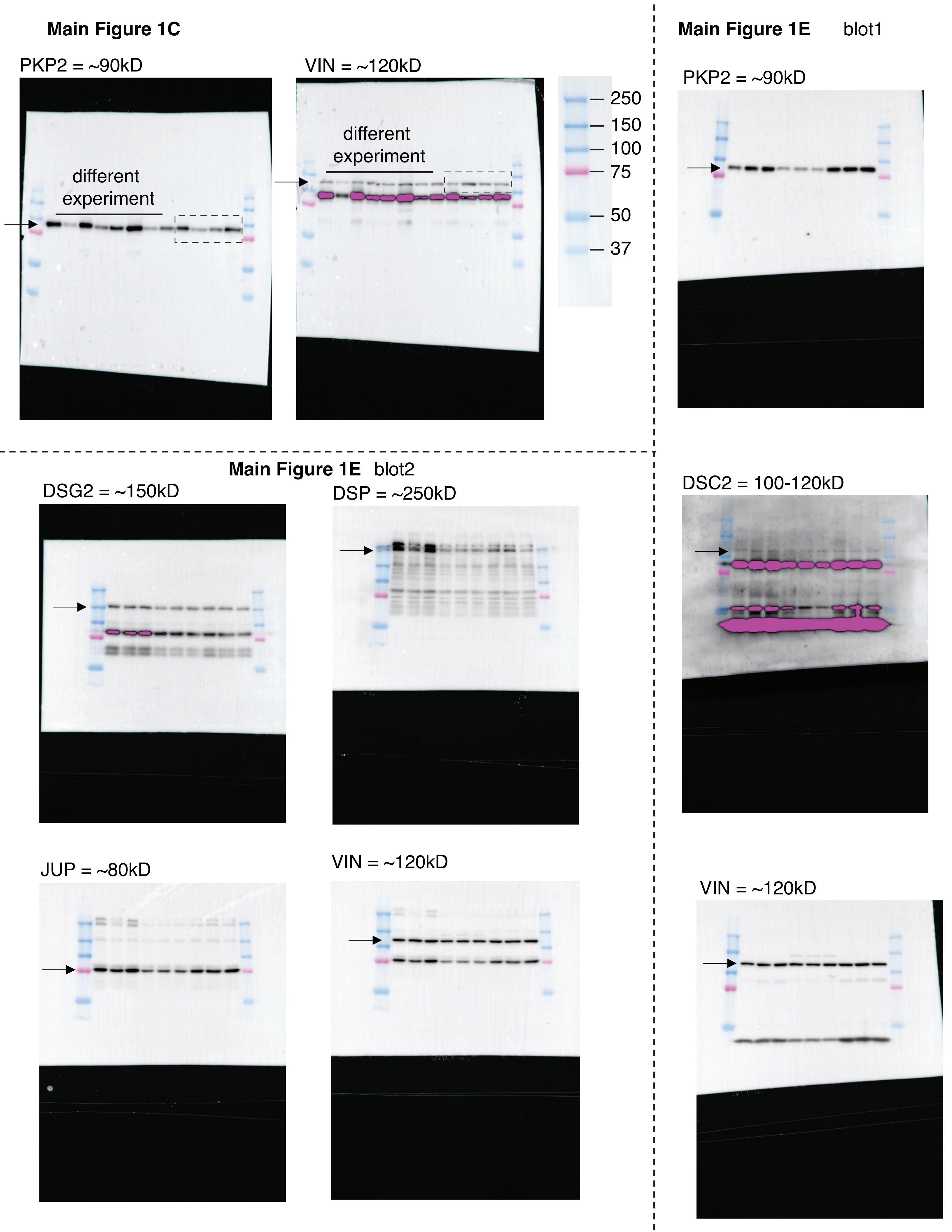

Main Figure 1C
PKP2 = ~90kD
different experiment
VIN = ~120kD
different experiment
250
150
100
75
50
37
Main Figure 1E blot1
PKP2 = ~90kD
DSC2 = 100-120kD
VIN = ~120kD
Main Figure 1E blot2
DSG2 = ~150kD
DSP = ~250kD
JUP = ~80kD
VIN = ~120kD
