# Supplementary material for: Therapeutic efficacy of AAV-mediated restoration of PKP2 in arrhythmogenic cardiomyopathy
Source: Nat Cardiovasc Res. 2023 Dec 7;2(12):1262–76. doi: 10.1038/s44161-023-00378-9 (PMC11041734; doi:10.1038/s44161-023-00378-9)
Supplement: Supplementary file 7 — Unprocessed western blot. [file 44161_2023_378_MOESM7_ESM.pdf]

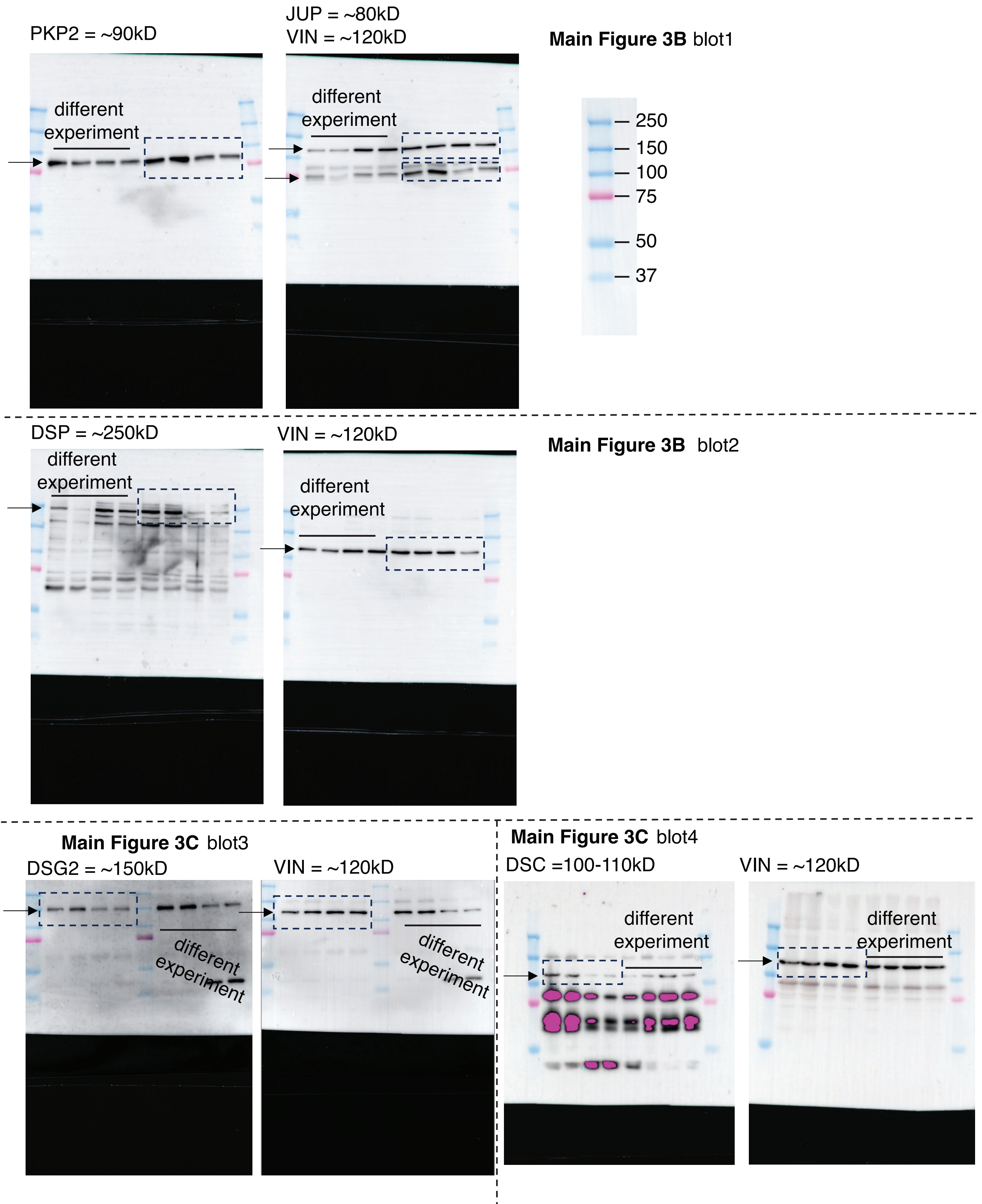
PKP2 = ~90kD
JUP = ~80kD
VIN = ~120kD
Main Figure 3B blot1
different experiment
different experiment
250
150
100
75
50
37
DSP = ~250kD
VIN = ~120kD
Main Figure 3B blot2
different experiment
different experiment
Main Figure 3C blot3
DSG2 = ~150kD
VIN = ~120kD
different experiment
different experiment
Main Figure 3C blot4
DSC =100-110kD
VIN = ~120kD
different experiment
different experiment
