# Supplementary figures and images for: Therapeutic efficacy of AAV-mediated restoration of PKP2 in arrhythmogenic cardiomyopathy
Source: Nat Cardiovasc Res. 2023 Dec 7;2(12):1262–76. doi: 10.1038/s44161-023-00378-9 (PMC11041734; doi:10.1038/s44161-023-00378-9)

Main Figure 4C

blot 1

PKP2 = ~90kD

DSP = ~250kD

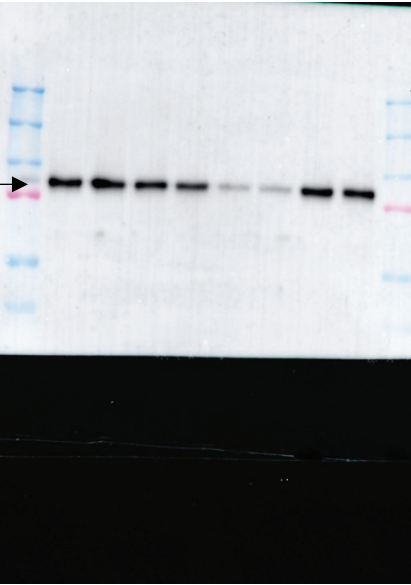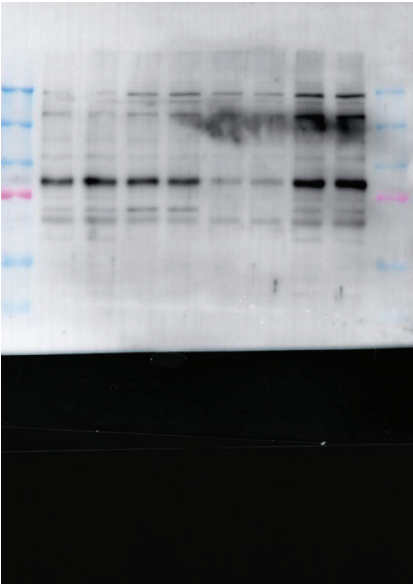

blot 2

DSG2 = ~150kD

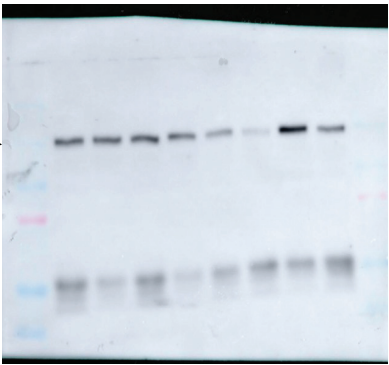

VIN = ~120kD

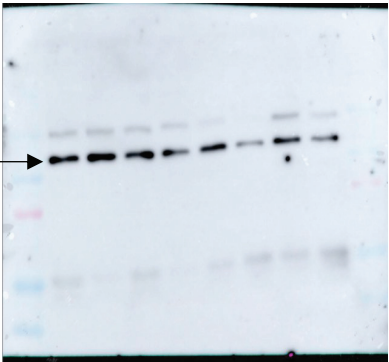

JUP = ~80kD

VIN = ~120kD

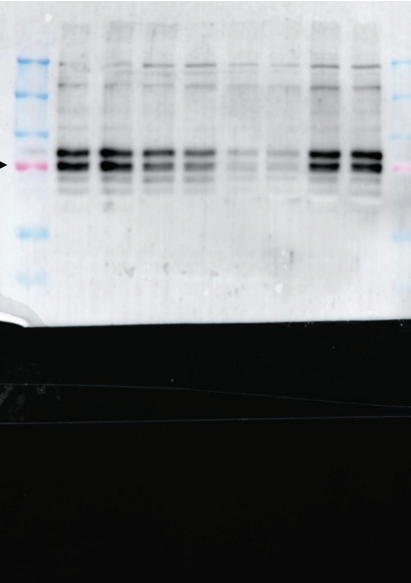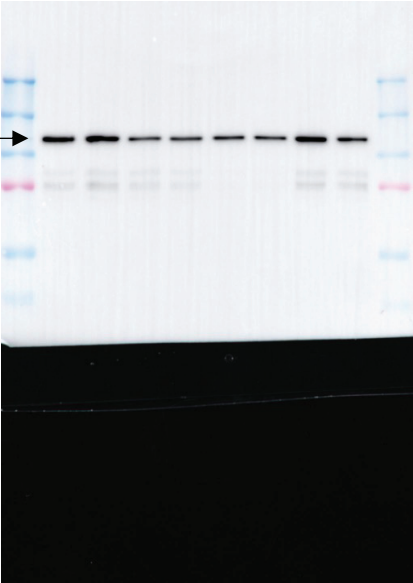

NCAD = ~150kD

$\alpha$ -CAT = ~98kD

$\alpha$ -TUB = ~50kD

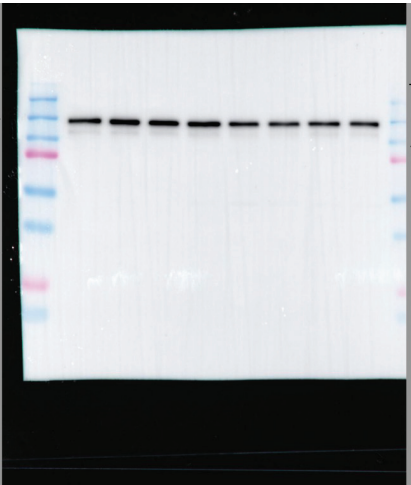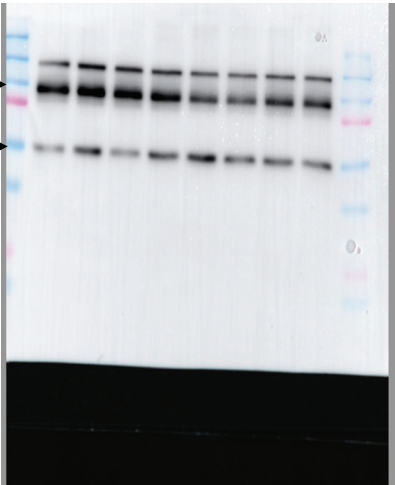

blot 3

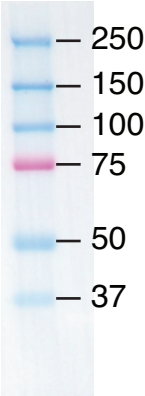

Supplement: Supplementary file 9 — Unprocessed western blot. [file 44161_2023_378_MOESM9_ESM.pdf]

**Main Figure 5D**

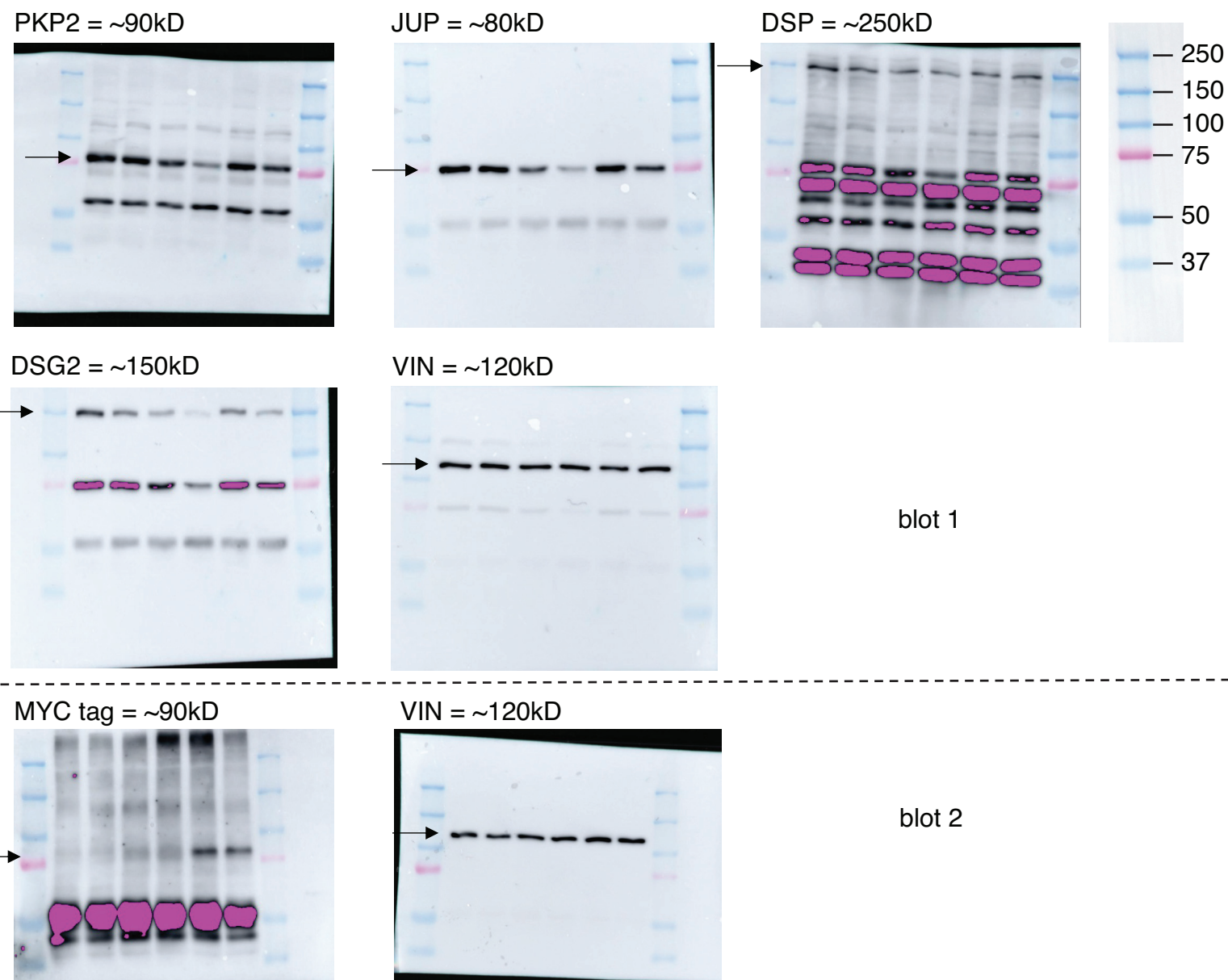

**Main Figure 5K**

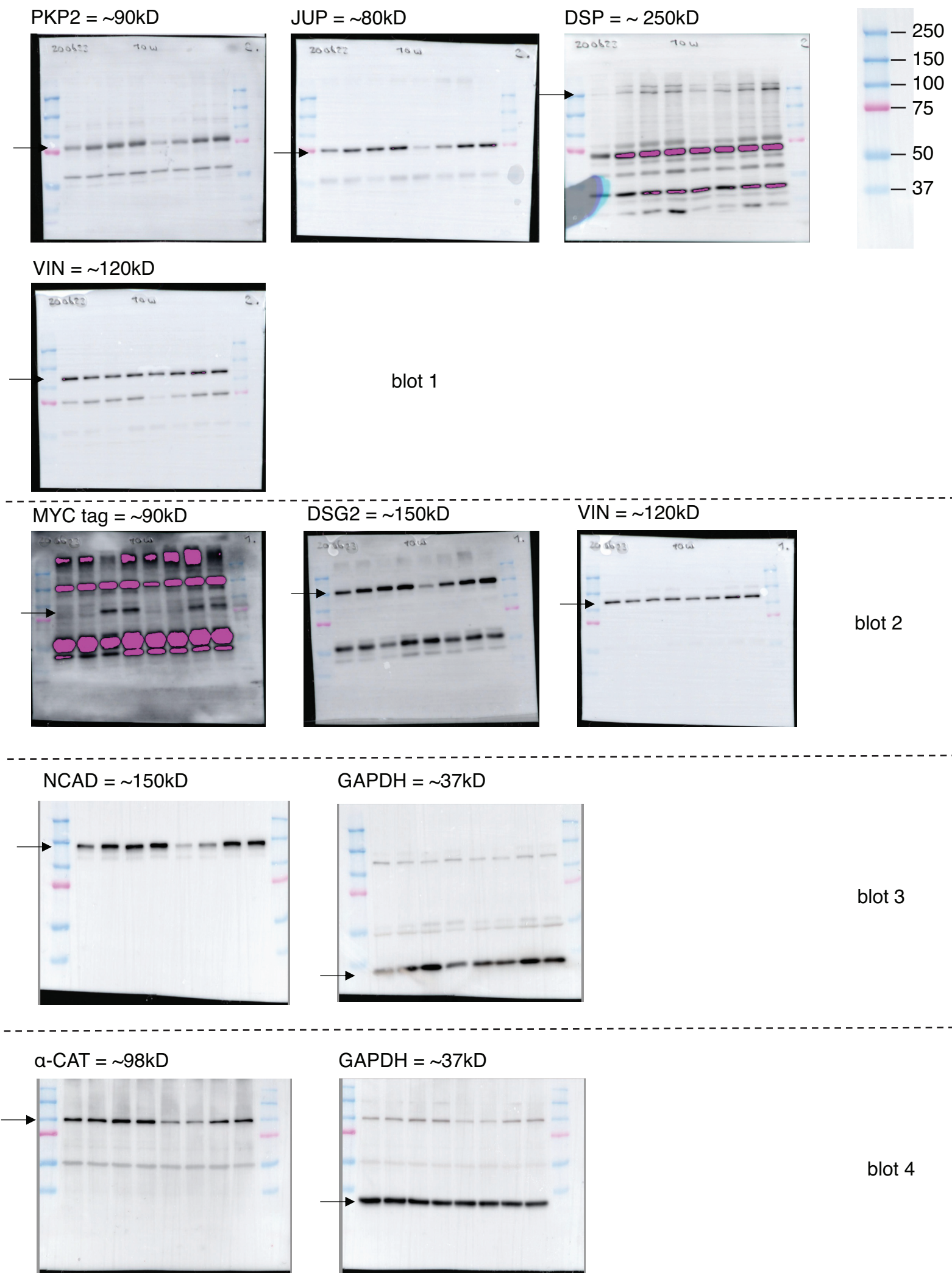

Supplement: Supplementary file 11 — Unprocessed western blot. [file 44161_2023_378_MOESM11_ESM.pdf]

Main Figure 6C

PKP2 = ~90kD

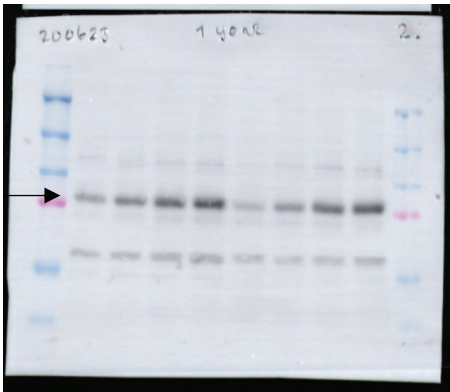

JUP = ~80kD

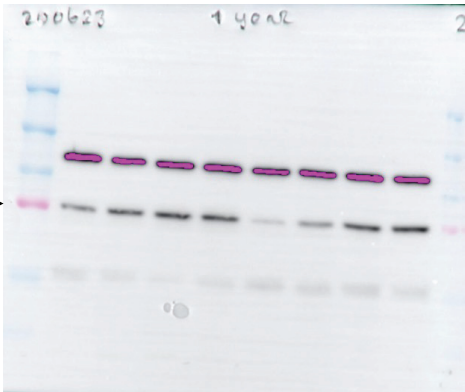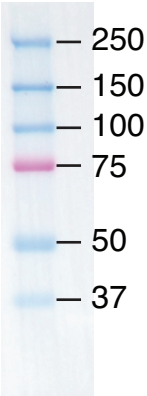

DSP = ~250kD

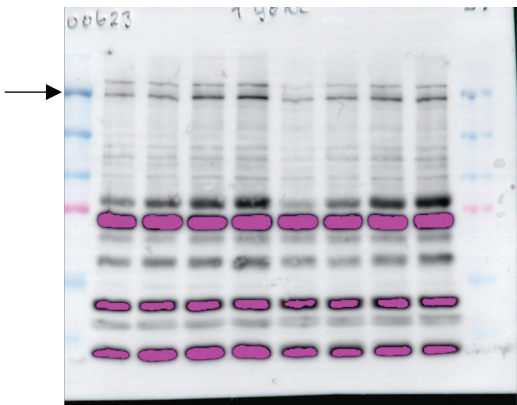

VIN = ~120kD

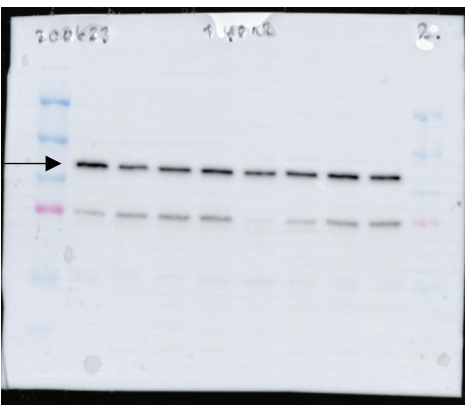

blot 1

MYC tag = ~90kD

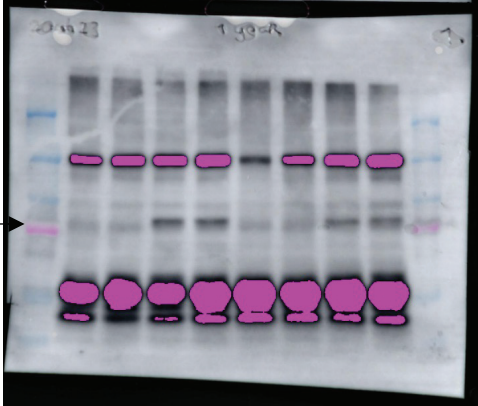

DSG2 = ~150kD

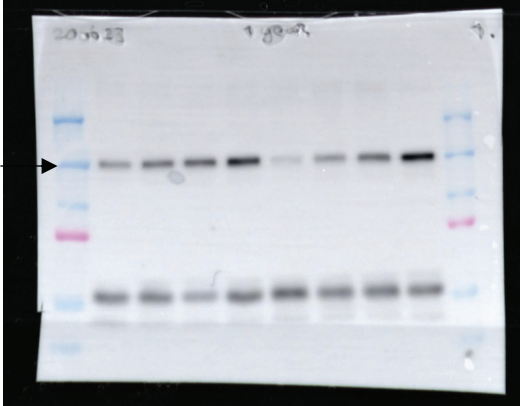

VIN = ~120kD

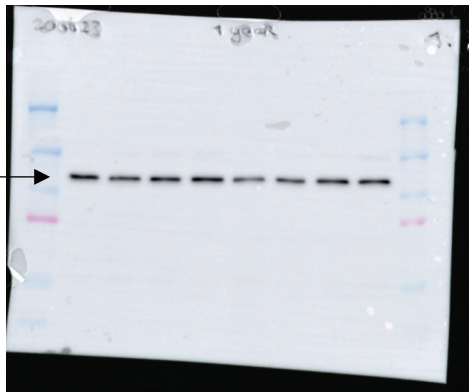

blot 2

Supplement: Supplementary file 13 — Unprocessed western blot. [file 44161_2023_378_MOESM13_ESM.pdf]

Extended Data Figure 2A

DSP = ~250kD

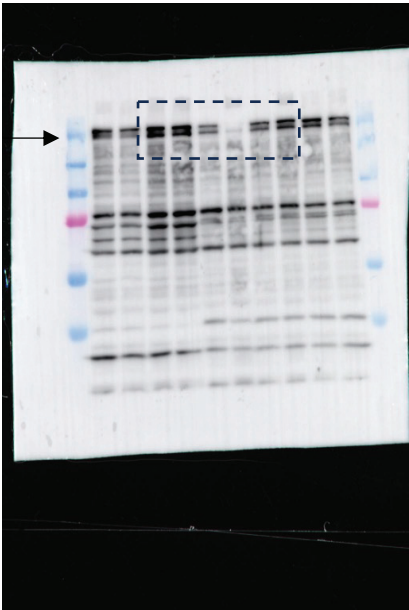

PKP2 = ~90kD

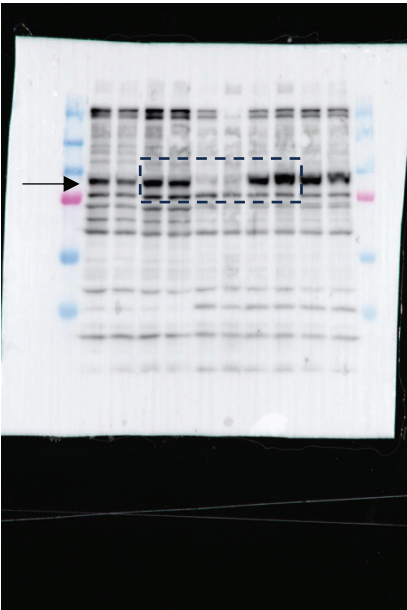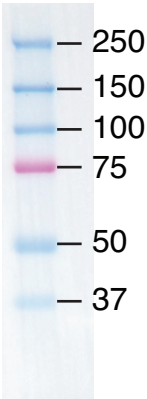

JUP = ~80kD

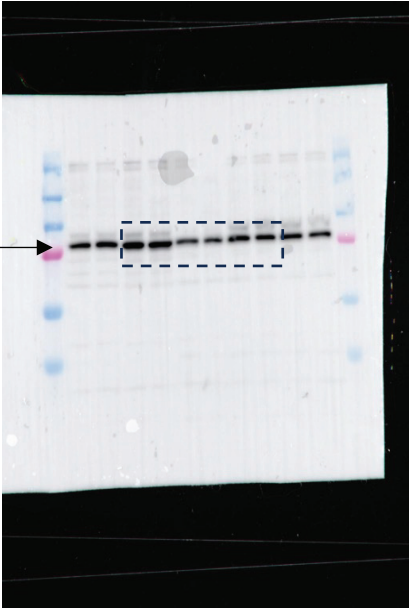

VIN = ~120kD

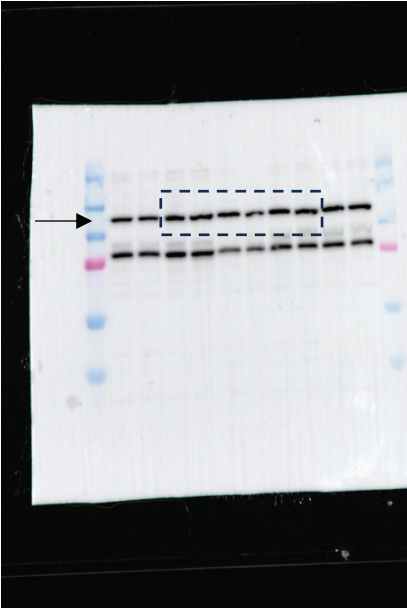

Supplement: Supplementary file 16 — Unprocessed western blot. [file 44161_2023_378_MOESM16_ESM.pdf]

Extended Data Figure 5A

DSC = 100-110kD

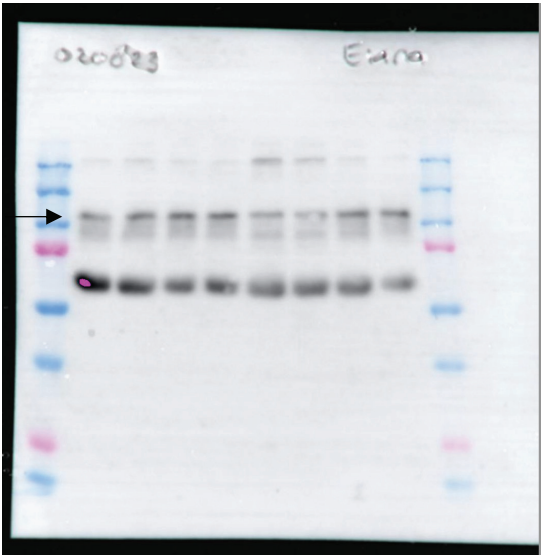

$\alpha$ TUB  $\approx$  50kD

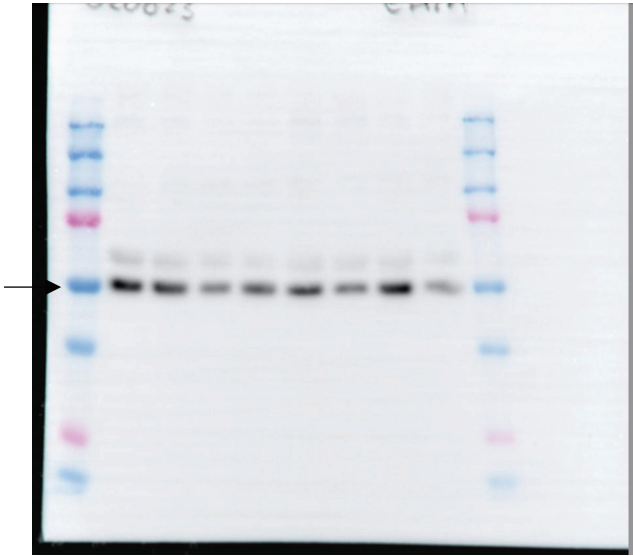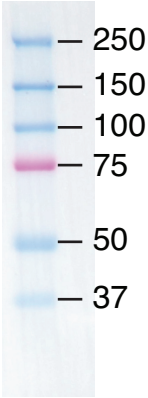

Supplement: Supplementary file 20 — Unprocessed western blot. [file 44161_2023_378_MOESM20_ESM.pdf]
