# Supplementary material for: Therapeutic efficacy of AAV-mediated restoration of PKP2 in arrhythmogenic cardiomyopathy
Source: Nat Cardiovasc Res. 2023 Dec 7;2(12):1262–76. doi: 10.1038/s44161-023-00378-9 (PMC11041734; doi:10.1038/s44161-023-00378-9)
Supplement: Supplementary file 22 — Unprocessed western blot. [file 44161_2023_378_MOESM22_ESM.pdf]

Extended Data Figure 6C

MYC tag = ~ 90kD

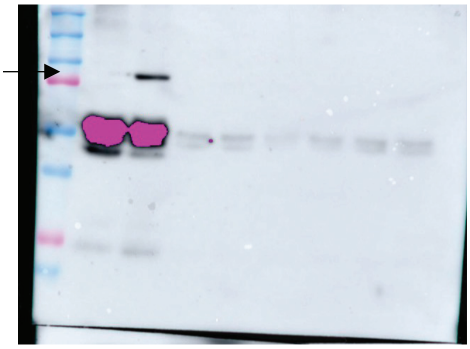

PKP2 = ~ 90kD

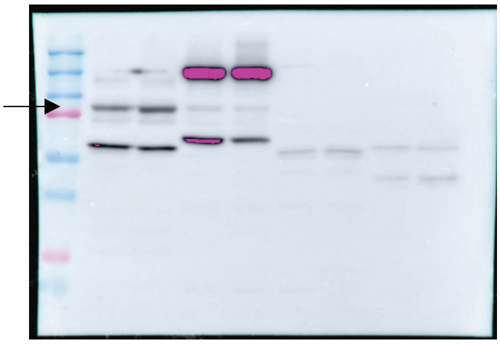

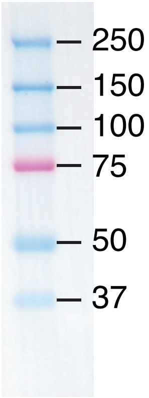

GAPDH = ~ 37kD

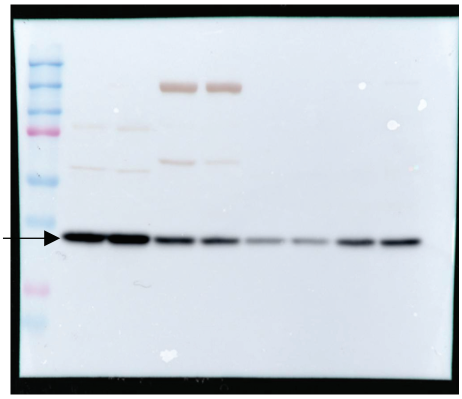

VIN = ~120kD

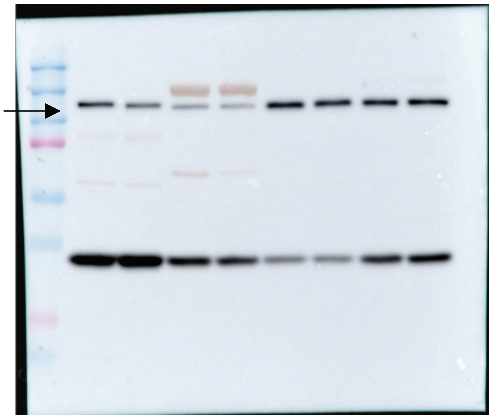

The lower band on VIN blot belongs to GAPDH which was initially run on the same blot as a loading control (see blot on the left). Even though both loading controls were comparable between the two genotypes on the same tissue, we chose to quantify with VIN to be consistent with our processing of the liver data (see below).

Extended Data Figure 6D

MYC tag = ~ 90kD

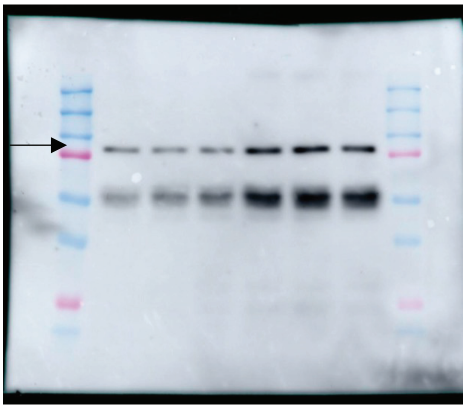

VIN = ~120kD

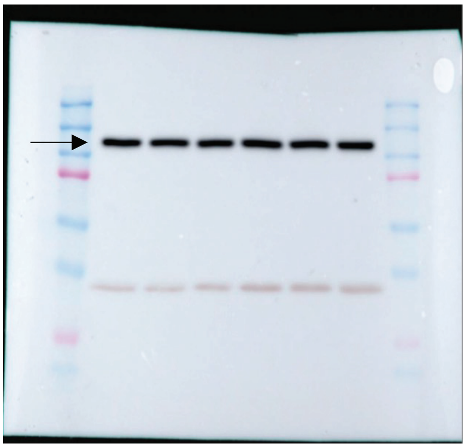

PKP2 = ~ 90kD

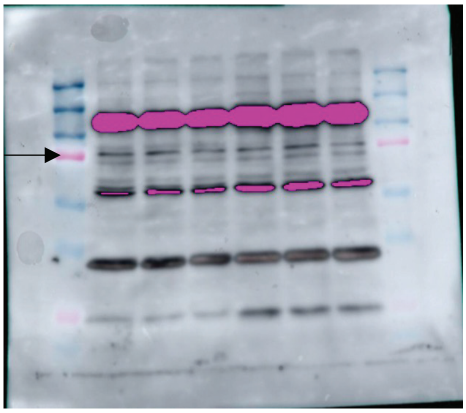

GAPDH = ~ 37kD

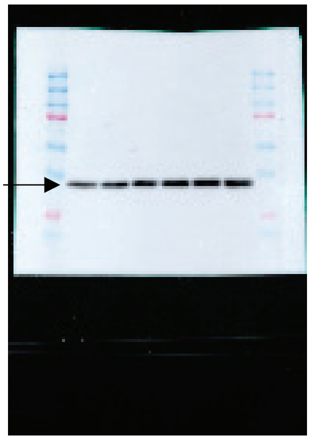

The lower band on VIN blot belongs to GAPDH which was initially run on the same blot as a loading control. However, VIN appeared more consistent between the two genotypes within the liver tissue, therefore we chose to proceed with this loading control for quantification.
On the right, the blot after GAPDH immunobloting.
