# Supplementary material for: Therapeutic efficacy of AAV-mediated restoration of PKP2 in arrhythmogenic cardiomyopathy
Source: Nat Cardiovasc Res. 2023 Dec 7;2(12):1262–76. doi: 10.1038/s44161-023-00378-9 (PMC11041734; doi:10.1038/s44161-023-00378-9)

Extended Data Figure 7A

DSC = 100-110kD

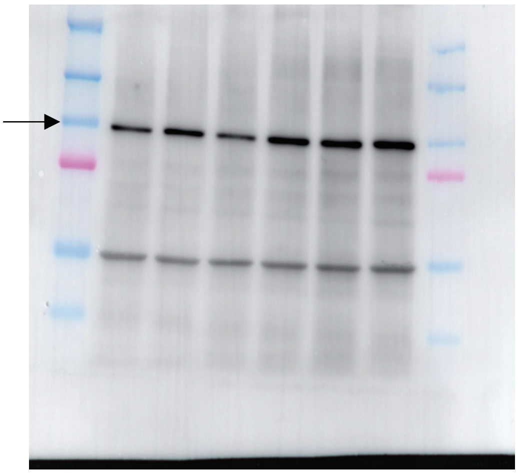

GAPDH = ~37kD

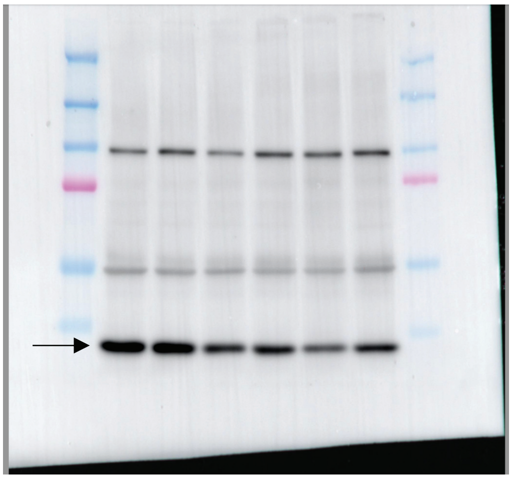

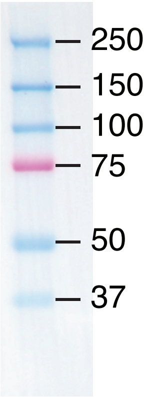

Extended Data Figure 7C

DSC = 100-110kD

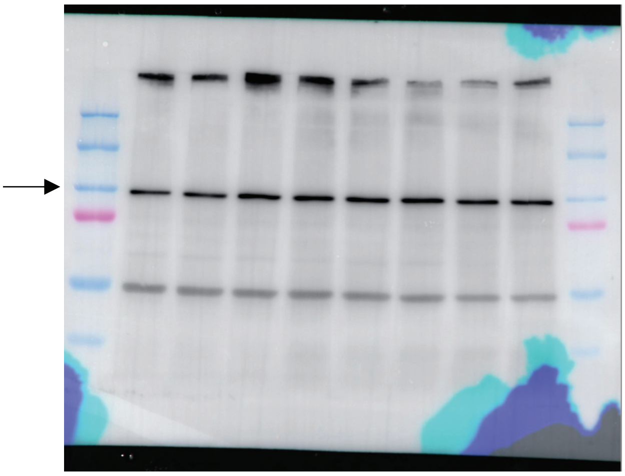

GAPDH = ~37kD

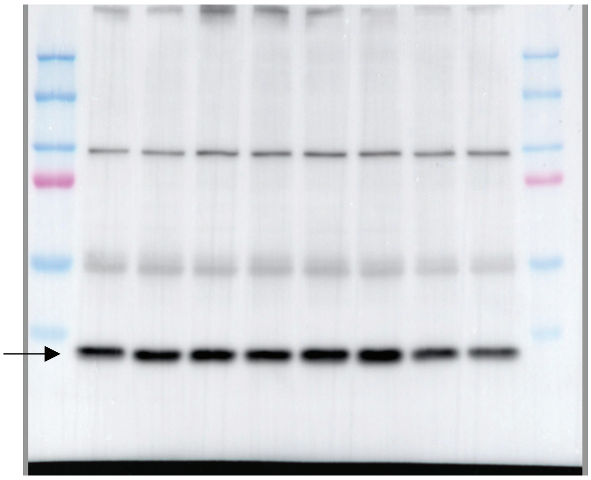

Extended Data Figure 7E

DSC = 100-110kD

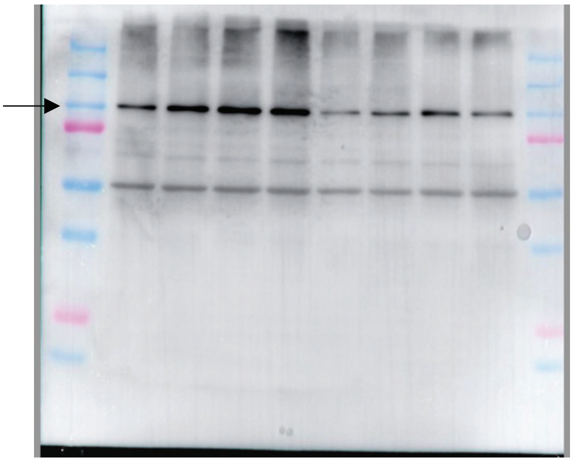

GAPDH = ~37kD

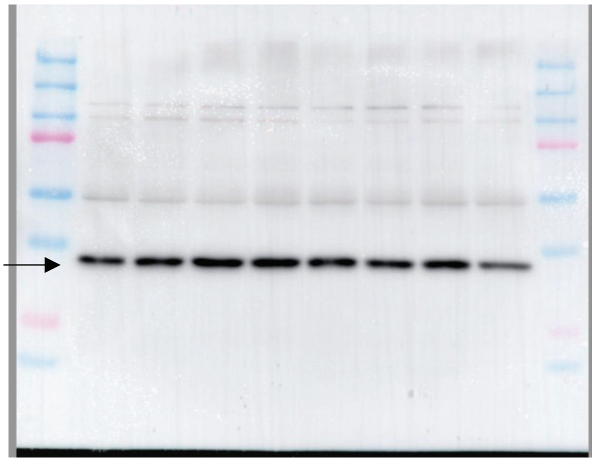

Supplement: Supplementary file 24 — Unprocessed western blot. [file 44161_2023_378_MOESM24_ESM.pdf]
